# Biobased Self‐Healing Thin Film Coatings Based on Poly (Itaconic Acid Esters)

**DOI:** 10.1002/cssc.202401499

**Published:** 2024-10-29

**Authors:** S. Charlotte Fischer von Mollard, Patrick Fesser, Michael Klein, Martin Köhler, Martin Schreiber, Frank Kamphuis, Stefan Zechel, Martin D. Hager

**Affiliations:** ^1^ Laboratory of Organic and Macromolecular Chemistry (IOMC) Friedrich Schiller University Jena Humboldtstr. 10 07743 Jena Germany; ^2^ Jena Center for Soft Matter (JCSM) Friedrich Schiller University Jena Philosophenweg 7 07743 Jena Germany; ^3^ ACTEGA Terra GmbH Industriestraße 12 31275 Lehrte Germany; ^4^ Helmholtz Institute for Polymers in Energy Applications Jena (HIPOLE Jena) Lessingstraße 12–14 07743 Jena Germany

**Keywords:** Biobased polymers, Poly(itaconic acid esters), Emulsion polymerization, Renewable polymers, Self-healing polymers, Polymer films

## Abstract

Paper used for packaging applications is often coated with thin polymer coatings to improve the properties, like printability and barrier properties, respectively. Today, these coatings are still often based on petroleum‐based polymers. In this study, the fabrication of biobased thin film coatings is described. Poly(itaconic acid ester)s, which are prepared by emulsion polymerization, are used as water‐based coatings for paper. The thermal properties of the polymers are tuned by the side chain of the monomers (diethyl itaconate *vs*. dibutyl itaconate). Different formulations based on the polymer emulsion and additives, like rheology modifiers, are prepared and their film formation is studied. The usage of a rheology modifier results in excellent film formation. These polymer coatings feature an additional function ‐ they are capable of self‐healing. The healing ability is studied in scratch healing tests, in which almost complete recovery can be observed after healing at 100 °C. Moreover, the restoration of optical properties/aesthetics is studied. In gloss measurements before and after damage as well as after a healing time the complete recovery of the gloss can be observed. Furthermore, the barrier properties against fat are studied.

## Graphical abstract

Paper coatings are important for improving the properties of packaging materials. Poly(itaconic acid ester)s are a promising sustainable alternatives to currently applied petroleum‐based polymers. Based on the poly(itaconic acid ester) emulsion, a thickener and ammonia, systems for paper coating were developed. These coatings feature self‐healing abilities in thin layers and the functional properties of the coating can be restored.

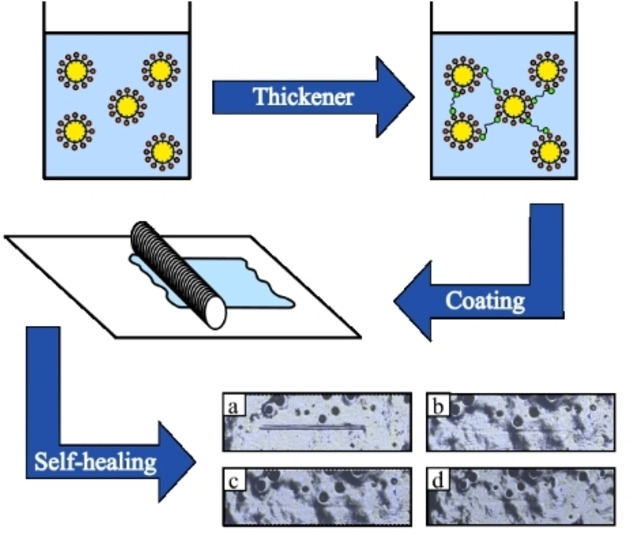

## 1 Introduction

Paper is a frequently used raw material in the packaging industry. Due to the hydrophilic properties of cellulose, it is difficult to control the penetration of moisture, oil, aromas and gases.[^1^, ^2^] In order to improve the (barrier) properties of paper, additives are added to the paper during production or they are coated afterwards.[^1^, ^2^] Polymers such as polyethylene (PE),^[3]^ poly(ethylene terephthalate) (PET),^[4]^ polystyrene (PS)^[5]^ or polyfluoralkyl substances (PFAS)^[1]^ are often used for these paper coatings. Due to the ecological point of view, there is an effort to use more sustainable polymers for paper coatings.^[1]^ Amongst the biobased alternatives studied, poly(lactic acid) has to be mentioned, which is already used today for such coatings.[^1^, ^6^, ^7^, ^8^] Further approaches for sustainable coatings are based on whey protein,^[9]^ shellac,^[10]^ alginates and soy protein.^[11]^

In the production of paper packaging, a sheet of paper is first coated with a primer or directly the printing is applied. Afterwards, an overprint varnish is applicated to protect the paper and the print. The actual paper packaging is cut out of the resulting sheet, folded and glued. When the packaging is folded, cracks may form in the coating, which impairs the barrier properties. This effect can be overcome by the utilization of self‐healing coatings.

Self‐healing polymeric coatings can be based on extrinsic or intrinsic self‐healing materials.[^12^, ^13^, ^14^] The former coatings are mostly based on embedded healing agents in microcapsules, which means that a relatively thick layer is required.[^14^, ^15^] Nevertheless, it has been implemented for applications as coatings for wood,^[16]^ aluminum^[17]^ or steel.^[13]^ For instance, the brand META Prime already sells self‐healing products for steel coatings.^[18]^ Intrinsic self‐healing coatings do not feature a strong limitation concerning the layer thickness, since they are mostly based on reversible interactions within the material.^[19]^ Consequently, they are advantageous for our aimed application as the layer thickness is significantly lower than 10 μm. In the literature there are some examples of self‐healing polymer coatings for paper, such as self‐healing polyurethanes^[20]^ as well as calcium carbonate microcapsules.^[21]^

Poly(itaconic acid esters) are already known for their self‐healing ability in the bulk.^[22]^ We could showed that a polymer consisting of diethyl itaconate (DEI) with 5% monoethyl itaconate (MEI) featured the best self‐healing ability. A healing efficiency of 99% could be achieved after 2 h at 90 °C in scratch healing tests using the bulk materials. Due to the limited solubility in water, this polymer can only be processed using organic solvents, like ethanol, which limits the practical usage. For a potential industrial application, it is advantageous if the polymers can be processed using aqueous formulations. Consequently, no flammable or toxic fumes are created when the polymer coating is applied on paper and subsequently dried. Polymer dispersions, which can be obtained from emulsion polymerization, are the material of choice.^[23]^ The optimization of the emulsion polymerization for itaconic acid esters has been reported before.^[24]^

**Figure 1:**
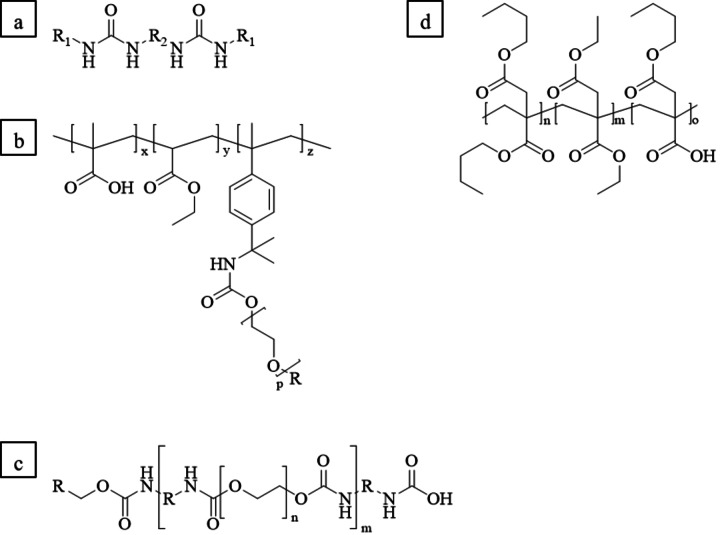
Structure examples for different rheology modifiers: **a**) Modified urea,^[26]^ **b**) HASE,[^28^, ^29^] **c**) HEUR.^[31]^ Furthermore, the structures of the polymers (**P1**: n:m:o=95 : 0 : 5, **P2**: n : m : o=0 : 95 : 5, **P3**: n : m : o=47.5 : 47.5 : 5) is depicted in **d**).

Considering the final formulation of the coating, various additives are added to improve processability and the final properties of the coating. For instance, rheology modifiers are used to increase the viscosity of the emulsion (Figure 1).^[25]^ The higher viscosity of the emulsions prevents both the film from contracting due to surface tension and from spreading out. In general, there are different types of rheology modifiers like modified ureas,^[26]^ hydrophobically modified ethoxylated urethanes (HEUR),^[25]^ or hydrophobically modified alkali swellable acrylates (HASE).[^27^, ^28^, ^29^] They all have in common that they have both hydrophilic and hydrophobic groups, which enables the micelles to form networks *via* hydrogen bonds. Furthermore, there are thickeners without any interaction with the polymer. One of them is laponite, a synthetic clay.^[30]^

The thickener Rheobyk‐7420 CA, used in this study, is a modified urea.^[32]^ The addition of the thickeners increases the interaction between the polymer particles, which results in the thickening effect.

## 2 Results and Discussion

### 2.1 Synthesis of the Polymer Dispersions

Aqueous polymer emulsions were prepared as the fabrication of the paper coatings should be based on an aqueous formulation due to a lower environmental impact and a better transability into application. For all emulsion polymerizations, a monomer mixture of the corresponding dialkyl itaconate (or a mixture of these monomers) and monobutyl itaconate (MBI) (95 : 5) was utilized. Overall, three different polymers were prepared, which differ in the used dialkyl itaconate. **P1** contains dibutyl itaconate (DBI), **P2** diethyl itaconate (DEI) and **P3** a 1 : 1 mixture of DBI and DEI (Figure 1). For the further tests, also a 1 : 1 mixture of **P1** and **P2** was prepared. The resulting molar masses of the polymers was determined by SEC‐measurements. The results are summarized in Table 1 and the graphs are depicted in the supporting information (Figures S7–S9).

**Table 1:** Results of the SEC‐measurements of the polymers.

Polymer	M_n_	M_w_	Ð^[a]^
	[g mol^−1^]^[a]^	[g mol^−1^]^[a]^	
**P1a**	21,100	110,300	5.20
**P2a**	8,800	18,600	2.12
**P3a**	71,400	201,500	2.82

[a] Determined *via* SEC measurements (THF, PMMA‐standard).

In general, the molar masses of the polymers are relatively low for polymers obtained by emulsion polymerizations. This finding can be explained by the poor polymerizability of itaconates.^[24]^

NMR measurements of the polymers **P1**, **P2** and **P3** proved the chemical structure of all synthesized copolymers. All signals could be assigned to the different groups in the polymers and no unconverted monomer could be observed (no vinyl protons were visible in the ^1^H NMR spectrum). The exact composition of the copolymers could not be determined by NMR spectroscopy, since the relevant signals of the different monomers (dialkyl‐ and monoalkylitaconates) are overlapping. All spectra are shown in Figures S1, S3 and S5.

Moreover, ionomers **Pxc** were prepared by addition of ammonia. These polymers were also analyzed by NMR spectroscopy in the same way as for the polymers. All signals can also be assigned in this case (see Figures S2, S4 and S6).

### 2.2 Thermal and Thermomechanical Properties

The thermal and thermomechanical properties of the polymers are important parameters for the self‐healing studies, which are described below. In our previous study, the thermal (DSC and TGA) as well as mechanical properties (DMTA, rheology, indentation) of the poly(itaconate)s have already been studied for copolymers containing MBI and DEI, comparable to **P2** in the present study.^[22]^ Nevertheless, all polymers and all ionomers were analyzed regarding the thermal properties and the results of the DSC and TGA measurements are summarized in Table 2. The corresponding measurement results are shown in the supporting information (SI, Figures S10–S27).

**Table 2:** Thermal properties of the polymers.

Polymer	*T*_g_^[a]^ [°C]	*T*_d_^[b]^ [°C]
**P1a**	7	303
**P1c**	5	304
**P2a**	34	307
**P2c**	53	304
**P3a**	32	310
**P3c**	31	302
**P1+P2c**	8 57	307

[a] Determined *via* DSC measurements during the 3^rd^ cycle with a heating rate of 10 K min^−1^. [b] Determined *via* TGA measurements with a heating rate of 10 K min^−1^.

In general, the DSC measurements reveal that the glass transition temperature of **P1** is lower than that of **P2**. Since **P1** is the copolymer of DBI and MBI and **P2** is the copolymer of DEI and MBI, this behavior was expected, since a similar behavior is known for other polymer classes such as acrylates.[^33^, ^34^] The *T*_g_‐value of the terpolymer **P3** is between that of **P1** and **P2**, which is an expected behavior for copolymers. The mixture of the polymers **P1** and **P2** has two glass transition temperatures, which are approximately the *T_g_*‐values of both polymers **P1** and **P2**, which indicated the presence of two different phases.

The TGA measurements revealed that all polymers have a decomposition temperature of around 300 °C (Table 2, SI Figures S19–S27). Therefore, the temperature used during processing and healing does not lead to any decomposition.

Furthermore, dynamic mechanical thermal analysis was performed for the polymers **Pxa** and the ionomers **Pxc**. Since the polymers are applied as coatings, the measurements were performed in plate‐plate geometry. All results are depicted in the supporting information (Figures S28–S35). The investigation of the polymers revealed a softening of the polymers and the ionomers. The polymers containing DEI feature a higher softening temperature compared to the DBI polymers. However, in several cases, the contact between the polymer and the plates of the rheometer is poor resulting in slightly noisy signals.

### 2.3 Formulation

The synthesized emulsions were directly used for the subsequent formulations without further purification. As control sample, the emulsions were used without any additive (series **Pxa**). As the viscosity of these emulsions is rather low, a rheology modifier was added resulting in the series **Pxb**. In order to neutralize the free carboxylic acid of the MBI, the polymer emulsions were brought to pH=9 with ammonia (series **Pxc**) and for the last series **Pxd** 1% Rheobyk‐7420 CA was added to the neutralized solutions (**Pxc**) and stirred in for 5 min. In Table 3 all formulations are summarized.

**Table 3:** Overview of the various formulations that were examined in the course of the study.

Polymer	Additive			
	Without additive	Rheobyk‐7420 CA	Ammonia	Ammonia+Rheobyk‐7420 CA
**P1**	**P1a**	**P1b**	**P1c**	**P1d**
**P2**	**P2a**	**P2b**	**P2c**	**P2d**
**P3**	**P3a**	**P3b**	**P3c**	**P3d**
**P1+P2**	**P1+P2a**	**P1+P2b**	**P1+P2c**	**P1+P2d**

### 2.4 Film Formation

The film‐formation was studied for all formulations prepared (see above). Pieces of paper were coated with a wire bar coater (wet film thickness of 10 μm and 100 μm) and the resulting polymer films were assessed optically after drying of the films. The formulations **P1a**, **P1b**, **P3a**, **P3b**, **P3c**, **P1+P2a** and **P1+P2b** do not form closed films and showed only a poor film forming ability. As detailed above, the viscosity of the polymer dispersions is rather low, consequently, series **Pxa** (*i. e*. without any additives) fails. The poor film formation of formulation series **Pxb** can be explained by the requirements of the rheology additive. This additive requires a neutral to slightly alkaline pH‐value in order to have a thickening effect. Therefore, two other formulation series were investigated featuring an alkaline pH‐value, which was obtained by the addition of ammonia. In this context, **Pxc** contains no and **Pxd** was prepared by the addition of a rheology additive. All formulations, which did not result in homogenous films were not utilized for the further studies. Generally, the polymer films based on polymers with DBI and MBI are slightly sticky. This is due to the lower glass transition temperature (*T*_g_) of **P1**.

### 2.5 Self‐Healing Tests

All formulations, which showed good film formation, have been utilized for the investigation of the self‐healing ability.

As classical characterization, scratch healing was studied, since this type of damage mostly occur for films and coatings. In order to quantify the healing effiency, tactile profile measurements were performed.^[22]^

**Figure 2:**
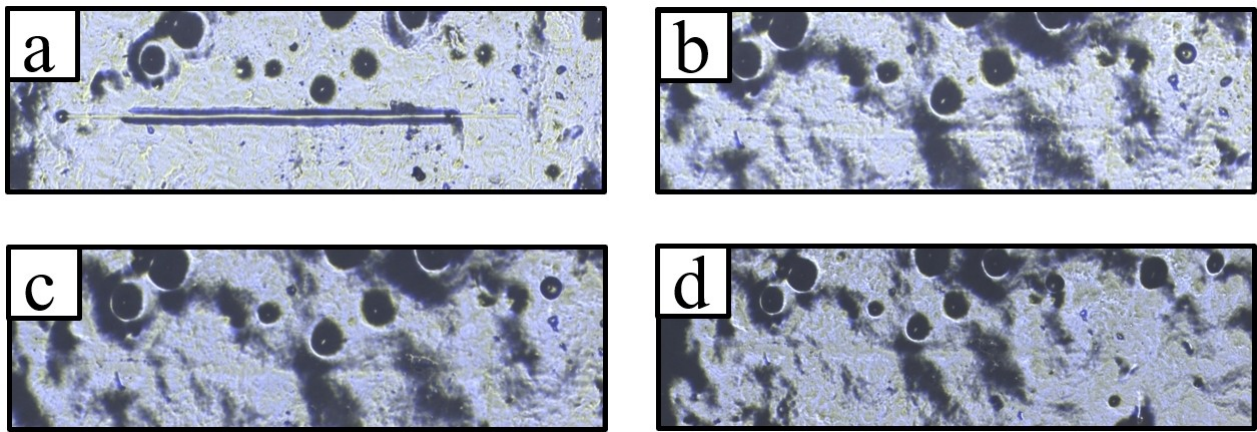
Analysis of the healing behavior of a coating of **P1+P2d** with a layer thickness of 200 μm. Different healing times are shown. In **a**), the crack can be seen in its initial state, in **b**) after 15 min, in **c**) after 1 h and in **d**) after 24 h (healing at 100 °C).

Previously, we had studied the self‐healing of poly(itaconate)s as bulk materials with a scratch tester.^[22]^ Therefore, this method was also applied for the coatings on paper. For the measurements, films with a layer thickness of 200 μm were applied to the paper, in order to obtain thick enough coatings. Otherwise, the indenter would damage the paper and not only the polymer coating. Furthermore, the indenter would bend the paper during scratching leading to an uneven surface preventing the analysis of the scratch volume before and after healing. In addition, the paper has a rough surface, which makes it difficult to detect the cracks on thinner films leading to the necessity of thicker films. The thicker films features bubbles/holes, which formed during the drying of the coating (Figure 2).

All coated papers were damaged over a length of 200 μm. The healing process was initiated by heating to 100 °C ‐ a temperature comparable to our previous study.^[22]^ Due to the deep scratches and the low material amount within the coatings compared to the bulk material, longer healing times were required. For example, in the case of **P1+P2d**, complete healing of the cracks can only be observed after 24 h (Figure 2).

The other samples performed similarly in the scratch healing tests (Figures S52–S61). As a general trend, it can be observed that the samples of **P1** tend to heal slightly better than those of **P2**, which can be attributed to the lower *T*_g_‐value leading to a higher degree of mobility during the healing process. Furthermore, the ionomers, which are the samples with ammonia (**Pxc** and **Pxd**) heal slightly better than the samples without ammonia (**Pxb**). The good healing ability of the ionomers is in accordance with our previous study.^[22]^ Interestingly, the holes derived from the bubbles do not vanish during the healing process indicating that the material is not completely flowable. A similar behavior was also previously observed in polymers based on reversible Diels‐Alder chemistry.^[35]^

**Figure 3:**
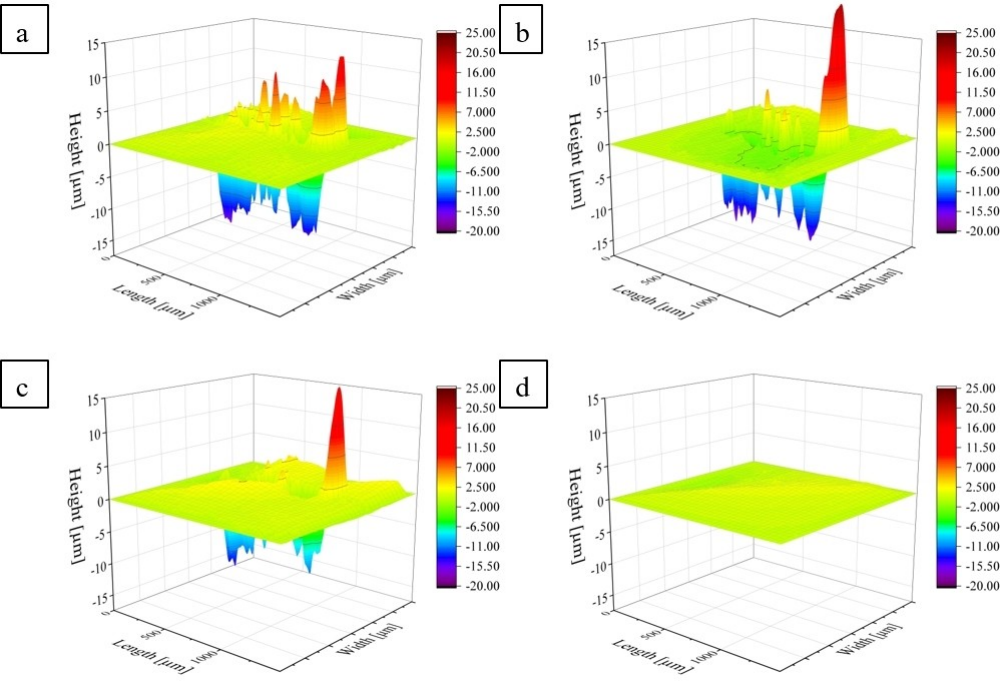
Quantification of the healing behavior of **P3a** featuring 3D‐plots of the profile at different times of self‐healing. In **a**), the crack can be seen in its initial state, in **b**) after 15 min, in **c**) after 1 h and in **d**) after 24 h at 100 °C.

Unfortunately, the quantification of the healing was not possible in thin films on paper due to the rough surface of the polymer films. Therefore, profile measurements were carried out for thicker materials. Exemplarily, the profile measurement of **P3a** is shown in Figure 3. The other measurements can be found in the SI (Figures S62–S67). The healing efficiency was calculated using the initial volume (*V*_I_) of the scratch and the volume after healing (*V*_H_) (Equation 1.
(1)

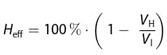




In general, the sample shows that the crack begins to close after just 15 min at 100 °C. However, according to calculations, the healing efficiency is −97%, as the polymer flows into the crack and, thus, forms a depression on the surrounding area. After 1 h, the surface has clearly leveled and *H*_eff_ is 24%. With a healing time of 24 h, the crack is no longer recognizable and *H*_eff_ is 89%. This schematic progression, from the first closing with a negative *H*_eff_ to the leveling of the surface, can be observed in all samples. Table 4 shows the measured values of the other measurements.

**Table 4:** Scratch healing of the bulk polymers with healing efficiencies after 15 min and 1 h as well as 24 h.

Sample	*H*_eff_ after 15 min [%]	*H*_eff_ after 1 h [%]	*H*_eff_ after 24 h [%]
**P1a**	15	23	14
**P1c**	−2	−1364	−1944
**P2a**	−2	−95	−41
**P2c**	−2	−638	−3
**P3a**	−97	24	89
**P3c**	42	29	99

The sinking of the surface can be observed in all samples. Therefore, the healing efficiencies cannot be fully evaluated. However, the pictures show that the cracks are becoming less deep. Thus, despite the partly negative *H*_eff_, it can be determined that the crack heals over time. A similar behavior was also observed for other polymers in literature, where the initial healing step resulted in a material flow but a broader scratch with negative healing values.[^36^, ^37^]

After the evaluation of the crack closure behavior, the function of the coating was studied as well as healability. As properties, the optical properties (*i.e*. aesthetics/gloss) and the barrier function were chosen. For these self‐healing tests, the polymer coatings have been damaged manually by driving over them with a steel sponge five times in horizontally and vertically leading to visible scratches on the surface.

In order to be able to quantify the healing of the optical properties/aesthetics, we decided to use the gloss of the coatings as a measure. Such an approach was described by Paquet *et al*. previously for the study of the self‐healing *via* gloss measurements.^[16]^ In contrast to their definition of the healing efficiency, the self‐healing efficiency in our study is calculated by dividing the value of the healed coating by the initial value of the sample. This calculation is based on the “classical” definition used for the calculation of healing efficiencies based on mechanical properties determined by tensile measurements.[^16^, ^22^] In the course of the study, the gloss values were determined at different angles, specifically at 20°, 60° and 85°. However, as the coatings are in the medium gloss range, only the measurement angle at 60° is discussed in more detail (Table 5); the other angles also show a similar trend and are listed in the SI (Tables S1–S6). The gloss values were normalized to the initial value for a better comparability.

**Table 5:** Gloss measurements at an angle of 60° (gloss values are normalized).

Sample [polymer, wet film thickness]	Before damaging [%]	After damaging [%]	After healing for 15 min at 100 °C [%]	After healing for 1 h at 100 °C [%]
**P1c** 10 μm	100±1	21±13	67±4	78±3
**P1d** 10 μm	100±1	61±6	99±4	102±3
**P2b** 10 μm	100±1	82±5	103±6	112±3
**P2c** 10 μm	100±8	83±8	122±6	124±5
**P2d** 10 μm	100±1	73±5	95±6	103±6
**P3b** 10 μm	100±4	40±8	94±4	99±2
**P3d** 10 μm	100±1	22±11	70±15	81±2
**P1+P2b** 10 μm	100±3	51±7	84±1	94±1
**P1+P2c** 10 μm	100±13	42±11	71±11	78±10
**P1+P2d** 10 μm	100±1	52±4	83±3	91±3
**P1c** 100 μm	100±3	24±14	96±1	101±1
**P1d** 100 μm	100±2	39±10	97±3	97±3
**P2d** 100 μm	100±1	59±3	80±1	84±1
**P3b** 100 μm	100±2	32±4	88±3	89±2
**P3d** 100 μm	100±2	32±7	80±5	82±7
**P1+P2b** 100 μm	100±2	49±14	85±9	97±10
**P1+P2c** 100 μm	100±2	33±11	68±6	75±3
**P1+P2d** 100 μm	100±1	48±3	93±1	106±1

In general, it can be seen that all polymer films feature a good healing ability. The samples **P1** have a higher healing efficiency after short healing times (15 minutes), as these are the samples with the lowest *T*_g_‐values. With these samples, a healing efficiency of approx. 70% (**P1c** 10 μm) to 100% (**P1d** 10 μm, **P1c** 100 μm, **P1d** 100 μm) can be achieved after just 15 minutes. One special case is **P2**. On the one hand, the gloss does not decrease as much when the sample is damaged (*e. g*., 73% for **P2d** 10 μm), which can be explained as **P2** features the highest glass transition temperature. This value is, in contrast to **P1** for example, above room temperature and, consequently, the polymers are less soft. On the other hand, the gloss values after healing are higher than the initial ones (up to 124% after 1 h for **P2c** 10 μm). This is caused by the fact that the **P2** samples form a very brittle and matt films. These films become shinier if they are annealed for a longer time, as the cracks in the mat surface are healed as well. The gloss values during the healing of the terpolymer **P3** do not differ significantly from the mixture of copolymers **P1+P2**. If, for example, **P3d** and **P1+P2d** with a layer thickness of 10 μm are compared, 70%–80% of the original value is restored after 15 min and 80%–90% after one hour. Overall, all polymer coatings showed good to excellent healing efficiencies for the restoration of the gloss. Consequently, the functional property can be restored.

Another important property of a paper coating is its barrier property, especially against grease.[^9^, ^38^] Generally, colored greases are used to study the barrier properties, since they penetrate the coating and color the paper underneath.^[39]^ The size of the color stains indicates the extent to which the oil/fat penetrates the coating. A similar test is ISO 16532–1, which works with palm fat and red paint. In general, the type of grease influences significantly how long it takes to break through the coating. Generally, turpentine or olive oil are more aggressive than palm oil and, therefore, penetrate the coatings more quickly.[^38^, ^40^]

**Figure 4:**
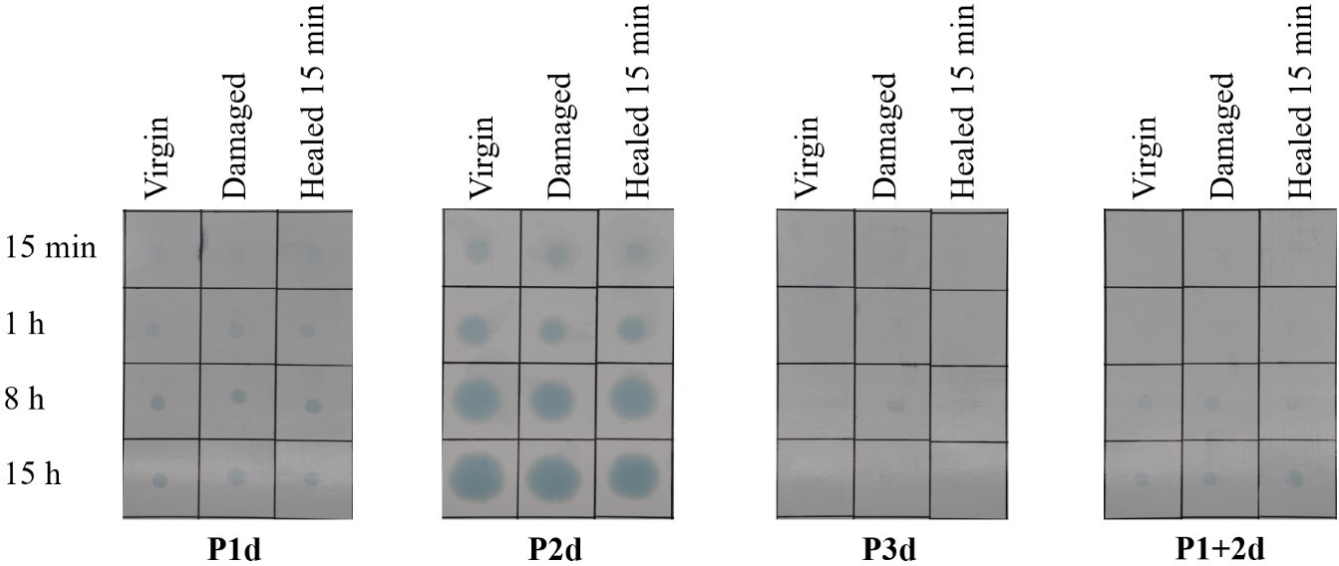
Barrier properties of the coatings against fat. The films are divided into grids; from left to right is the undamaged film, the damaged film and the film after 15 min curing at 100 °C. From top to bottom, the time dependency of the penetration in the paper is shown after 15 min, 1 h, 8 h and 15 h.

For the barrier tests, the formulations **P1d**, **P2d**, **P3d** and **P1+P2d** with a layer thickness of 10 μm were examined (Figure 4). The **Pxd** series was selected since it forms closed films across all **Px** and is therefore well comparable. The undamaged coating, the damaged coating and the coating after 10 min healing at 100 °C were investigated, whereby the grease had 15 min, 1 h, 8 h and 15 h to soak into the paper. A quantification was performed based on the colored area according to literature.^[41]^

Only **P1d** shows a slight coloration (ca. 3% of the area, see also Table S7) of the paper after 15 hours, whereby it makes no difference whether the sample was virgin, damaged or healed. For the terpolymer as well as the mixture of both polymers, **P3d** and **P1+P2d**, no to very little coloration of the paper could be detected at all (0% and 3%, see also Tables S9 and S10). The barrier test also shows that **P2** has a rather brittle surface. After a short time (even after 15 minutes), the paper under the polymer film turns blue indicating the ingress of the fat into the paper (ca. 40%, see Table S8).

In general, no difference can be seen between the undamaged films, the damaged films and the healed films (Figure 4). The damage with the steel sponge seems not to be strong enough to reduce the barrier properties of the films, even though the thickness of the films is very low. In case of **P2d**, the healing ability of the polymers does not lead to a sufficient flow of the material, which could potentially close the cracks of the more brittle coating **P2d**. This finding is slightly in contrast to the gloss measurements, where higher values of the healed sample could be detected. However, this behavior can be explained by the fact that only smaller scratches are healed, which increases the gloss value, but are not sufficient enough to result in a better barrier behavior.

## 3 Conclusions

Poly(itaconates) are promising candidates for self‐healing paper coatings. Within this study, we could show that self‐healing coatings can be obtained from aqueous polymer emulsions based on itaconates. However, the utilization of pure emulsions does not result in any film formation on paper (**P1a**, **P2a**, **P3a**, **P1+P2a**). In general, the best film‐forming properties were achieved by adding thickener and ammonia.

These coatings were studied with respect to their scratch healing behavior. Hereby, good self‐healing abilities even in very thin layers of 10 μm could be revealed. Scratch tests for thicker materials revealed that cracks can be healed and high healing efficiencies of up to 99% can be obtained. The restoration of functional properties could be demonstrated by gloss measurements, which enables the restoration of the aesthetics. The initial value could be restored even within short healing times. No influence of damage and crack closure could be determined on the barrier against fat, as superficial damage to the coating is not enough to impair the barrier properties.

One disadvantage to be solved in future studies is that the emulsions had to be processed promptly after mixing with the thickener, as they begin to precipitate within a few days. This may be due to the fact that the thickener leads to too strong interactions between the particles.

## 4 Experimental Section/Methods

### 4.1 Materials

Rheobyk‐7420 CA (Byk), ammonia solution 25% (Carl Roth), Sodium dihydrogen phosphate (NaH_2_PO_4_, Fluka), monobutyl itaconate (MBI, Sigma Aldrich), dibutyl itaconate (DBI, Sigma Aldrich), 2,2′‐azobis[2‐(2‐imidazolin‐2‐yl)propane] dihydrochloride (VA‐044, TCI) and sodium dodecyl sulfate (SDS, TCI) were used as received. Diethyl itaconate (DEI, TCI, stabilized with TBC) was purified over a column with neutral alumina (Molekula). Performa White^TM^ GC‐1 by Stora Enso were used as substrate for the coatings. It has a thickness of 325 μm and a grammage of 220 g/m^2^.

### 4.2 Instrumentation

#### 4.2.1 NMR Measurements

One‐dimensional nuclear magnetic resonance spectra (^1^H‐NMR) were recorded at 298 K using a Bruker (Rheinstetten, Germany) AC 300 (300 MHz). The NMR spectra were measured relative to the solvent signal of chloroform.

#### 4.2.2 SEC Measurements

Size exclusion chromatography was performed on a Shimadzu 10er series equipped with a DGU‐14 A degasser, a CBM‐20 A controller, a LC‐10AD vp pump, a SIL‐10AD vp autosampler and a CTO‐10 A vp oven, a PSS SDV guard/linear M column with 5 μm particle size and a separation range of 400–1,000,000 g mol^−1^. The measurement temperature was 30 °C with a flow rate of 1 mL min^−1^, tetrahydrofuran (THF) as eluent and PMMA as standard. As refractive index detector (RID) a RID‐10 A and as UV detector (UVD) a SPD‐10AD VP were used.

#### 4.2.3 DSC Measurements

The DSC measurements were not carried out directly on the emulsions. In order to measure the polymer, the pure emulsion was dialyzed in water (3 solvent changes) and THF (8 solvent changes). The resulting polymer was dried. To enable measurement of the ionomers, the polymer was also neutralized with ammonia. The measurements were then carried out on the on a Netzsch DSC 204 F1 Phoenix instrument (Selb, Germany) under a nitrogen atmosphere with a heating rate of 20 K min^−1^ (first and second cycle) and 10 K min^−1^ (third cycle).

#### 4.2.4 TGA Measurements

The samples for the TGA measurements were prepared like the samples for the DSC measurements. The thermogravimetric analysis (TGA) was carried under normal atmosphere with a heating rate of 10 K min^−1^ using a Netzsch TG 209 F1 Iris (Selb, Germany).

#### 4.2.5 Rheology/DMTA

The rheology measurements were performed according to a literature procedure^[42]^ and the sample preparation was performed similar to the DSC preparation. A MCR 302e rheometer from Anton Paar (Graz, Austria) was utilized using the convection oven device CTD 450. The samples were measured with a disposable plate‐plate measuring setup (D‐CP/PP25, Anton Paar (Graz, Austria)). The sample gap was set between 0.2 and 1.0 mm. The software RheoCompassTM V1. 30. 1064‐Release 64‐bit (Anton Paar, Graz, Austria) was applied for operating the rheometer as well as for analysis. The data was exported as txt‐files and evaluated and processed with OriginPro 2020 (OriginLab Corporation, Northampton, MA, USA).

#### 4.2.6 Scratch Tester Measurements

In order to examine the properties of the polymer films in more detail, circles with a diameter of 3 cm were cut out and glued to epoxy cylinders with a height of 1 cm. Furthermore, measurements in the bulk were performed. Therefore samples, prepared like in the DSC measurements, were hot pressed (5 min at 100 °C at about 2 t) in a special manufactured mold and embedded in epoxy resin. Afterwards the samples were grinded by sand paper (P80 to P3000). The measurements were performed on the Anton Paar Micro scratch tester MST3 on a STeP 4 platform using an established procedure.^[43]^ The instrument was equipped with 50 μm Rockwell B−E052 indenters and the optical images were taken with the lenses MPlan N 5×/0.10/FN22 and MPlan N 20×/0.40/FN22. For the panorama measurements one scratch with a length of 1000 μm was made (3000 μm min^−1^, 3 mN) and for the profile measurements 15 scratches with a length of 1000 μm were overlaid (30000 μm min^−1^, 300 mN). The profile measurement is carried out using a Rockwell K‐108 indentor. For this purpose, the sample is rotated by 90° and scanned every 20 μm with a load of 5 mN and a speed of 200 μm min^‐1^.

#### 4.2.7 Gloss Measurements

The gloss measurements were carried out with a micro‐TRI‐gloss from BYK‐Garder GmbH (Geretsried, Germany). The measuring angles are 20°, 60° and 85°. The polymer coatings were measured at four different spots. These were then damaged manually over a large area horizontally and vertically with a steel sponge by driving over them five times in both directions. The paper coatings were then cured in the oven at 100 °C for 15 min and for further 45 min (total 1 h).

#### 4.2.8 Testing of the Fat Barrier Properties

The test contains a 1 : 1 ratio of palm oil and margarine and sudan blue 670 (Sigma Aldrich). Four droplets of this mixture were applicated separately on the coated paper. After 15 min, 1 h, 8 h and 15 h the drops were wiped of and the coloring of the paper was visually evaluated.

To quantify the fat barrier, the coated cardboard was divided into 12 squares with an edge length of 4 cm. Four squares were prepared at a time. The undamaged coating was used first, followed by the damaged coating and then the damaged coating that had been healed at 100 °C for 15 minutes. 0.1 ml of grease was applied to the center of the squares and removed after 15 min, 1 h, 8 h and 15 h. Subsequently, the front and back of the paper were photographed, as shown in Figures S68 and S69. The blue colored area was analyzed using ImageJ. For this purpose, the edge length of the squares of 4 cm was used as a reference. As the contrast between the colored and uncolored areas was not great enough for automatic detection, the areas were analyzed manually. They were outlined by hand and the area was determined using the software ImageJ. This was carried out for all front sides and, in the case of **P2d**, also for the reverse side. The proportion of the colored area in relation to the total area of the squares was then determined. The results are summarized in the supporting information, Tables S7–S11.

#### 4.2.9 Synthesis of the Polymer Emulsions

The emulsions were synthesized as described in previous work.^[24]^ To shortly summarize, in a 150 mL sulfonation flask with stirrer shaft water, NaH_2_PO_4_ (0.025 g), VA‐044 (0.225 mmol, 0.072 g) and sodium dodecyl sulfate (SDS) (1 g) were dissolved. The solution was degassed for 1 h and then a solution of 25 mL of the diester with 5% of MBI was added under stirring. Subsequently, the resulting solution was stirred at 60 °C for 6 h. In order to be able to compare the properties of the coatings, three different emulsions were synthesized. The different calculations can be seen in Table 6.

**Table 6:** Compositions of the emulsions for the polymerizations.

Polymer	Diester	Monoester	Ratio (Diester:Monoester)
**P1**	DBI	MBI	95 : 5
**P2**	DEI	MBI	95 : 5
**P3**	DBI+DEI	MBI	47.5 : 47.5 : 5

The polymers were characterized by NMR spectroscopy and SEC measurements and the following results were obtained:

**P1**: ^1^H NMR (300 MHz, CDCl_3_) *δ*=0.96 (CH_3_ butyl), 1.40–1.60 (CH_2_ butyl), 2.37–2.61 (CH_2_ itaconyl), 3.98 (CH_2_ butyl) ppm.

SEC (THF, PMMA‐standard): M_n_=21,100 g/mol; M_w_=110,300 g/mol; Ð=5.20.

**P2**: ^1^H NMR (300 MHz, CDCl_3_) *δ*=0.95 (CH_3_ butyl), 1.25 (CH_3_ ethyl), 1.39–1.86 (CH_2_ butyl), 2.37–2.65 (CH_2_ itaconyl), 3.76 (CH_2_ butyl), 4.06 (CH_2_ ethyl) ppm.

SEC (THF, PMMA‐standard): M_n_=8,800 g/mol; M_w_=18,600 g/mol; Ð=2.12.

**P3**: ^1^H NMR (300 MHz, CDCl_3_) *δ*=0.94 (CH_3_ butyl), 1.25 (CH_3_ ethyl), 1.64–1.86 (CH_2_ butyl), 2.36–2.67 (CH_2_ itaconyl), 3.78 (CH_2_ butyl), 4.00 (CH_2_ ethyl) ppm.

SEC (THF, PMMA‐standard): M_n_=71,400 g/mol; M_w_=201,500 g/mol; Ð=2.82.

Furthermore, a mixture of **P1** and **P2** was prepared to compare the properties with **P3**. The mixture was calculated to a 1 : 1 ratio according to the molar amount of the polymer in the emulsion.

#### 4.2.10 Preparation of the Coating Solutions

Starting from the emulsions, various coating solutions were prepared (Table 7).

**Table 7:** Additives for different coating solutions.

Polymer	Additive			
	Without additive	Rheobyk‐7420 CA	Ammonia	Ammonia+Rheobyk‐7420 CA
**P1**	**P1a**	**P1b**	**P1c**	**P1d**
**P2**	**P2a**	**P2b**	**P2c**	**P2d**
**P3**	**P3a**	**P3b**	**P3c**	**P3d**
**P1+P2**	**P1+P2a**	**P1+P2b**	**P1+P2c**	**P1+P2d**

On the one hand, the pure emulsion without any additive were used (**Pxa**), for **Pxc** the pH‐value of the solution was brought to pH=9 by the addition of ammonia. Solutions with the indices **b** and **d** were obtained by the addition of 1 wt% of Rheobyk‐7420 CA to the series **Pxa** and **Pxc**, respectively. Due to interactions with the thickener, the emulsions precipitate after a few days. Therefore, the emulsions were processed timely.

#### 4.2.11 Preparation of the Polymer Films

The coating solutions were prepared as mentioned above and coated manually with a wire bar coater (Byk 200 mm, 10 μm and 200 mm, 100 μm wet film thickness) to Performa White^TM^ GC‐1 paperboard. Afterwards the coatings were dried at 100 °C for 15 min in a convection oven. The polymer films were investigated optically.

## Conflict of Interests

The authors declare no conflict of interest.

5

## Supporting information

As a service to our authors and readers, this journal provides supporting information supplied by the authors. Such materials are peer reviewed and may be re‐organized for online delivery, but are not copy‐edited or typeset. Technical support issues arising from supporting information (other than missing files) should be addressed to the authors.

Supporting Information

## Data Availability

The data that support the findings of this study are available from the corresponding author upon reasonable request.
