# Supplementary material for: Biobased Self‐Healing Thin Film Coatings Based on Poly (Itaconic Acid Esters)
Source: ChemSusChem. 2024 Oct 29;18(3):e202401499. doi: 10.1002/cssc.202401499 (PMC11789992; doi:10.1002/cssc.202401499)
Supplement: Supplementary file 1 — Supporting Information [file CSSC-18-e202401499-s001.pdf]

ChemSusChem

Supporting Information

Biobased Self-Healing Thin Film Coatings Based on Poly (Itaconic Acid Esters)

S. Charlotte Fischer von Mollard, Patrick Fesser, Michael Klein, Martin Köhler, Martin Schreiber, Frank Kamphuis, Stefan Zechel, and Martin D. Hager*

Supporting Information

Biobased self-healing thin film coatings based on poly(itaconic acid esters)

*S. Charlotte Fischer von Mollard, Patrick Fesser, Michael Klein, Martin Köhler, Martin Schreiber, Frank Kamphuis, Stefan Zechel, Martin D. Hager**

S. C. Fischer von Mollard, P. Fesser, M. Klein, Dr. S. Zechel, Dr. M. D. Hager
Laboratory of Organic and Macromolecular Chemistry (IOMC),
Friedrich Schiller University Jena, Humboldtstr. 10, 07743 Jena, Germany
Jena Center for Soft Matter (JCSM), Friedrich Schiller University Jena. Philosophenweg 7, 07743 Jena, Germany
E-mail: martin.hager@uni-jena.de

M. Köhler, Dr. M. Schreiber, F. Kamphuis
ACTEGA Terra GmbH, Industriestraße 12, 31275 Lehrte, Germany

Dr. M. D. Hager
Helmholtz Institute for Polymers in Energy Applications Jena (HIPOLE Jena),
Lessingstraße 12-14, 07743 Jena, Germany

Table of content

1. NMR measurements 3

2. SEC measurements 6

3. Differential scanning calorimetry (DSC) 8

4. Thermogravimetric analysis (TGA) 13

5. Dynamic mechanical thermal analysis (DMTA) 18

7. Self-healing of the coatings 34

8. Self-healing of the bulk 39

10. Barrier properties of the coatings against fat 46

1. NMR measurements

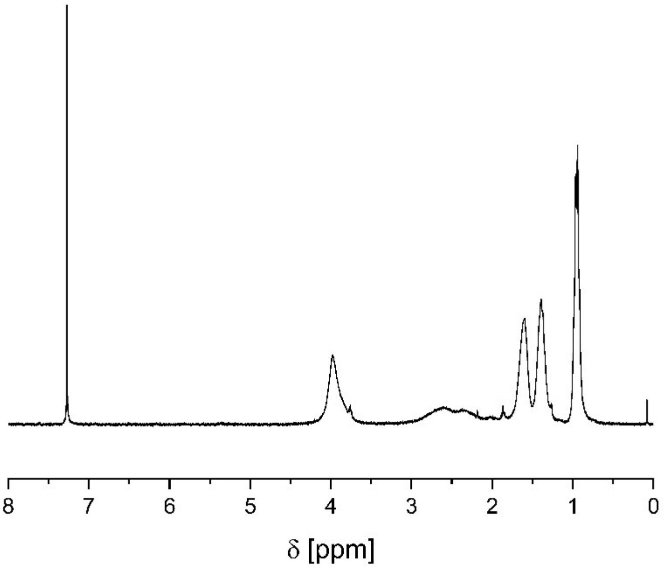

Figure S1. ^{1}H NMR spectrum of **P1a** (300 MHz, $CDCl_3$).

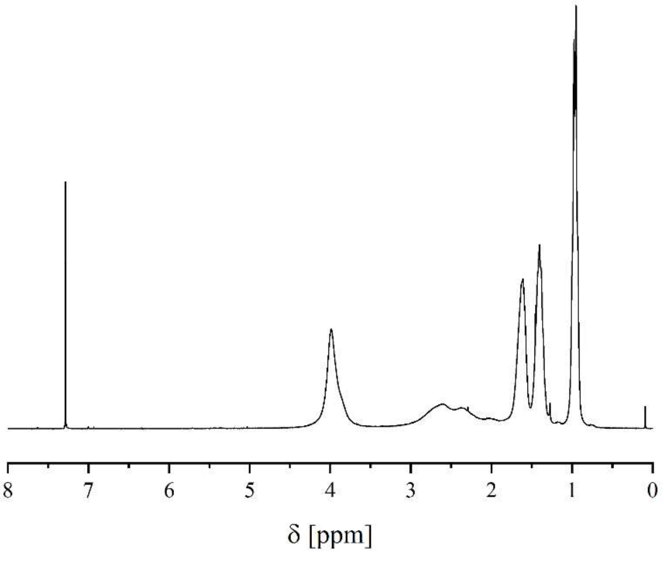

Figure S2. ^{1}H NMR spectrum of **P1c** (300 MHz, $CDCl_3$).

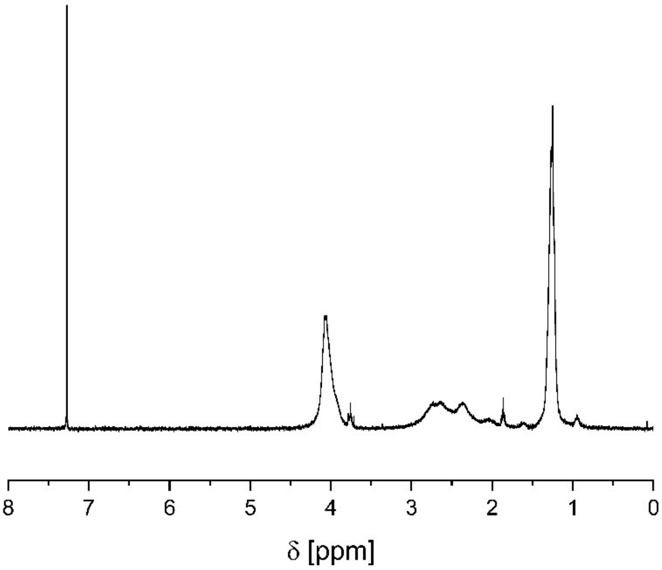

Figure S3. ^{1}H NMR spectrum of **P2a** (300 MHz, $CDCl_3$).

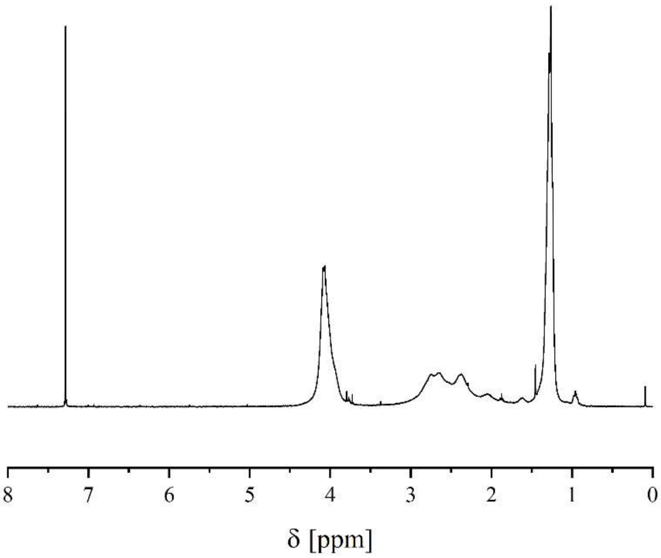

Figure S4. ^{1}H NMR spectrum of **P2c** (300 MHz, $CDCl_3$).

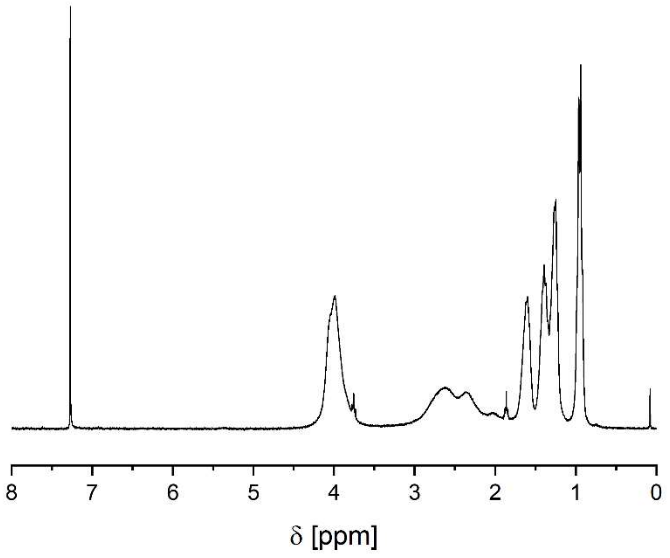

Figure S5. ^{1}H NMR spectrum of **P3a** (300 MHz, $CDCl_3$).

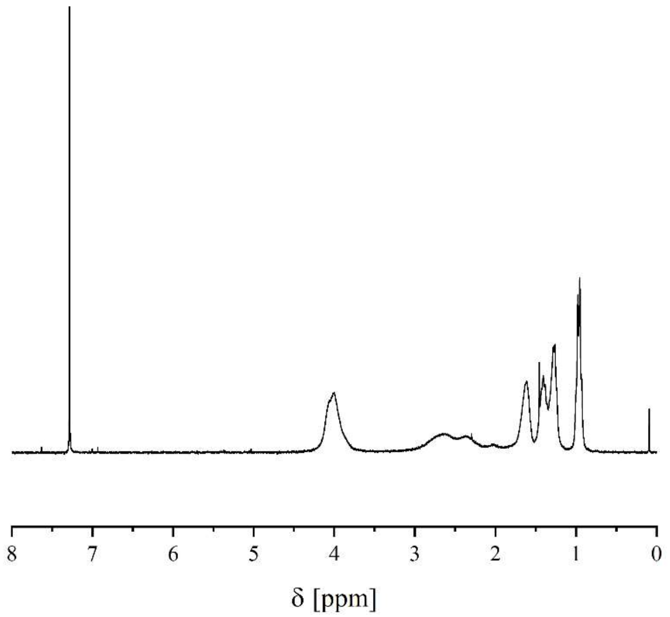

Figure S6. ^{1}H NMR spectrum of **P3c** (300 MHz, $CDCl_3$).

2. SEC measurements

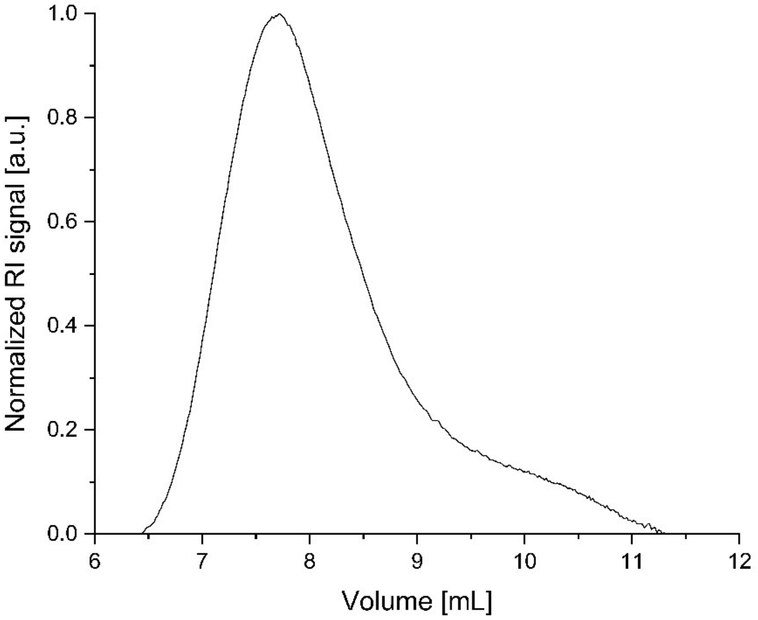

Figure S7. SEC curve of **P1a** (eluent: THF, PMMA-standard).

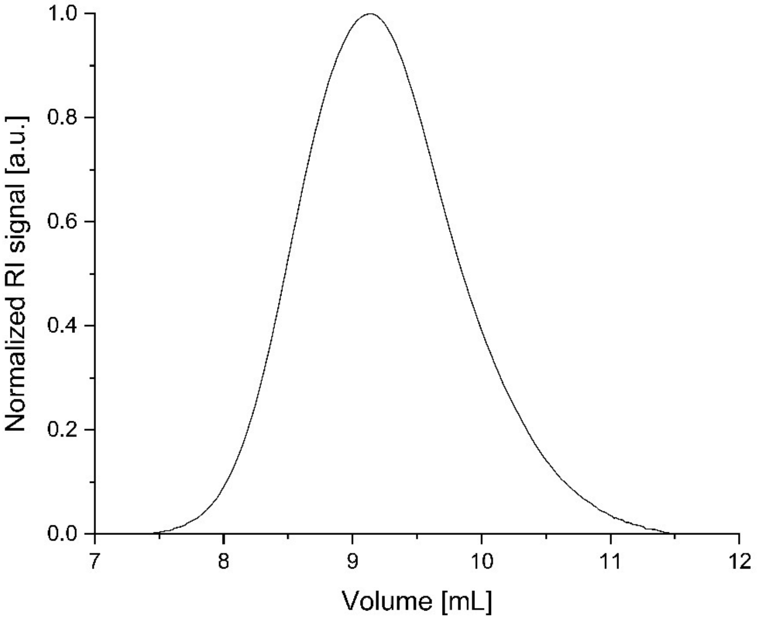

Figure S8. SEC curve of **P2a** (eluent: THF, PMMA-standard).

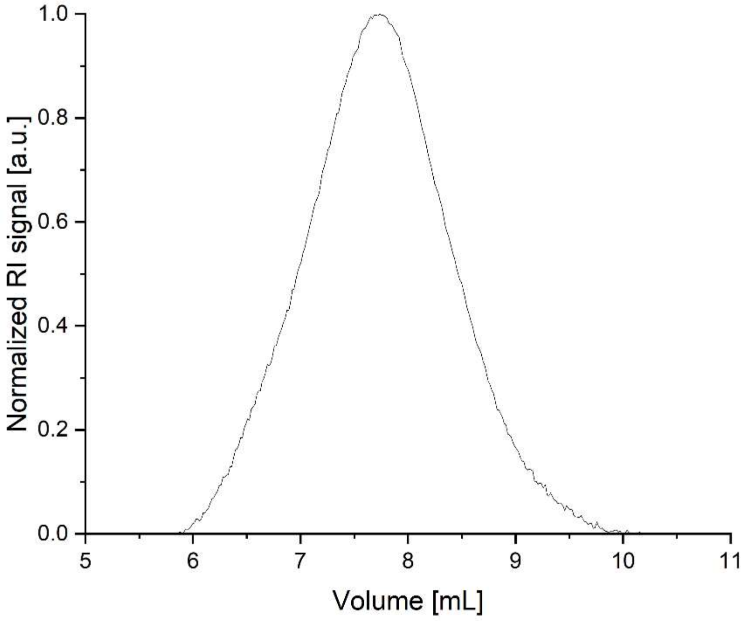

Figure S9. SEC curve of **P3a** (eluent: THF, PMMA-standard).

3. Differential scanning calorimetry (DSC)

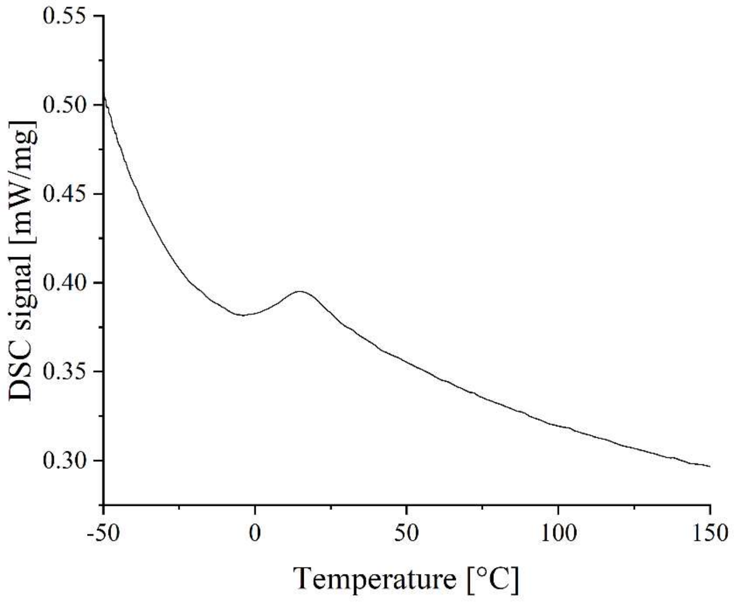

Figure S10. DSC-curves of the third cycle of polymer **P1a** with a heating rate of 10 K min^{-1}.

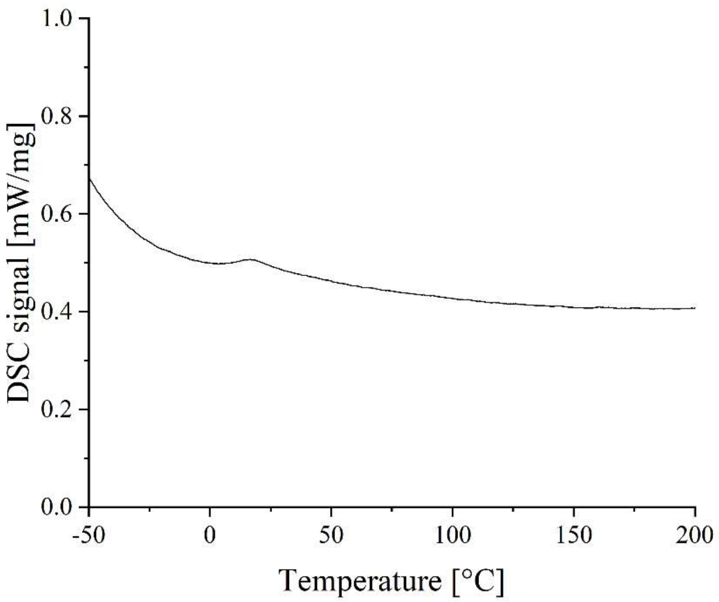

Figure S11. DSC-curves of the third cycle of polymer **P1c** with a heating rate of 10 K min^{-1}.

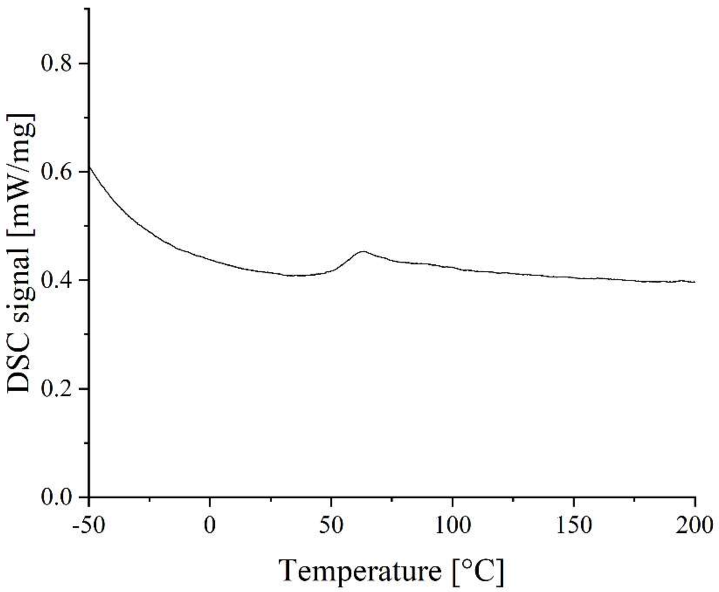

Figure S12. DSC-curves of the third cycle of polymer **P2a** with a heating rate of 10 K min^{-1}.

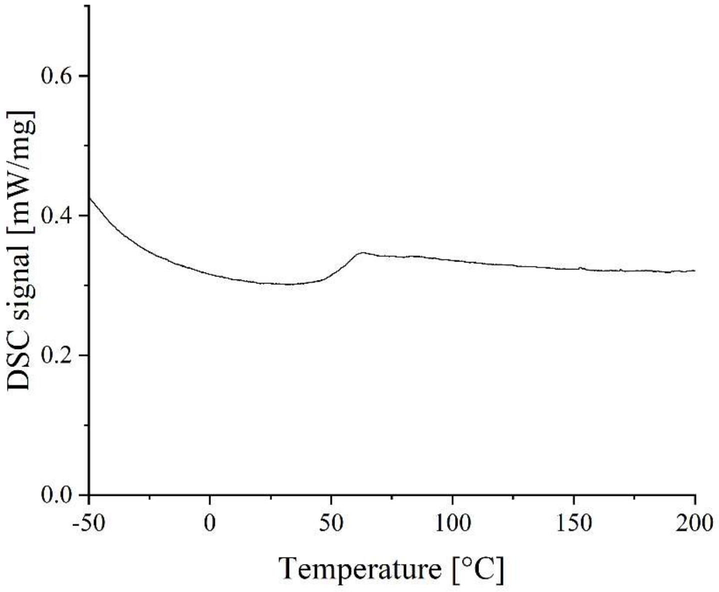

Figure S13. DSC-curves of the third cycle of polymer **P2c** with a heating rate of 10 K min^{-1}.

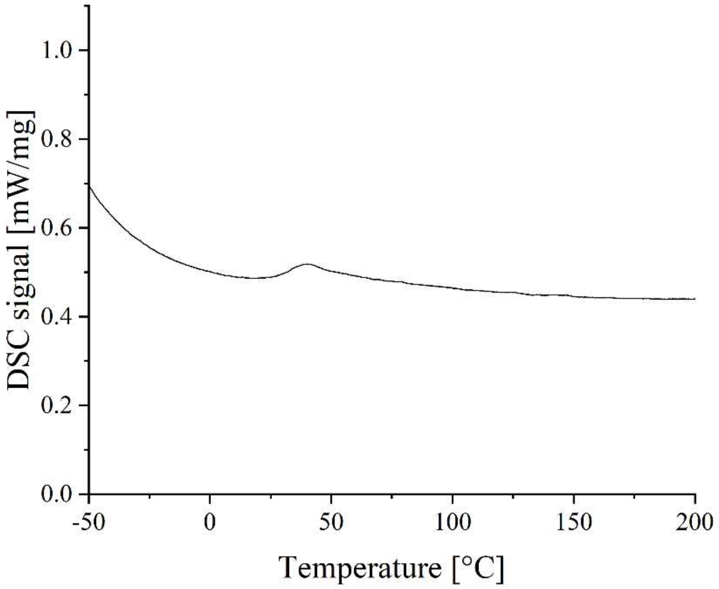

Figure S14. DSC-curves of the third cycle of polymer **P3a** with a heating rate of 10 K min^{-1}.

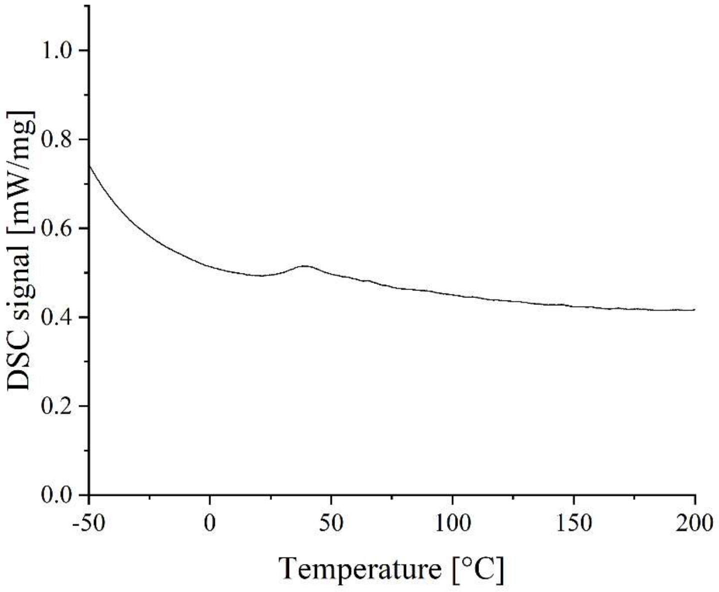

Figure S15. DSC-curves of the third cycle of polymer **P3c** with a heating rate of 10 K min^{-1}.

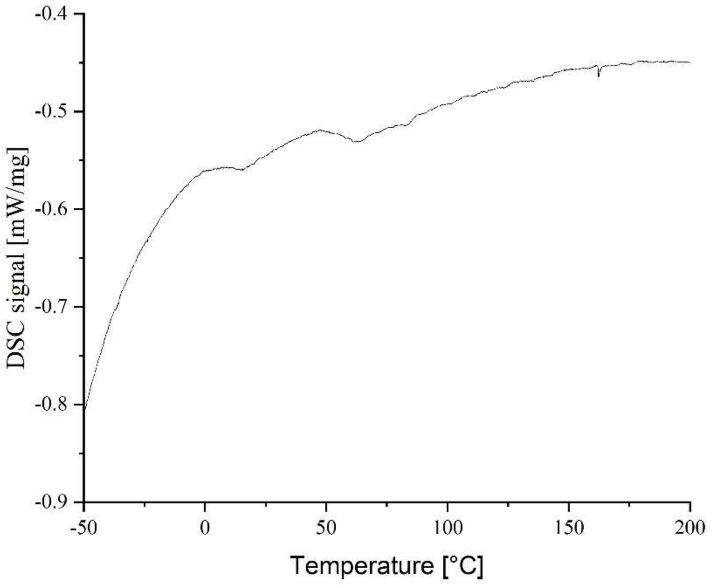

Figure S16. DSC-curves of the third cycle of polymer **P1+P2a** with a heating rate of 10 K min^{-1}.

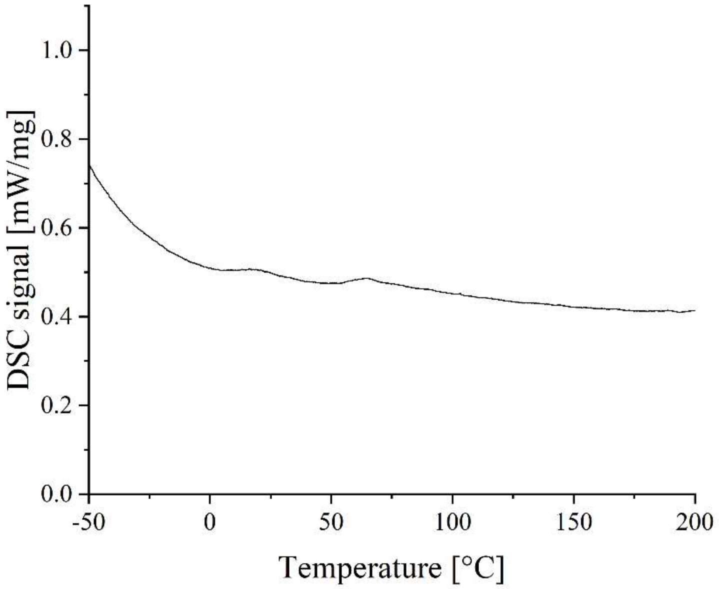

Figure S17. DSC-curves of the third cycle of polymer **P1+P2c** with a heating rate of 10 K min^{-1}.

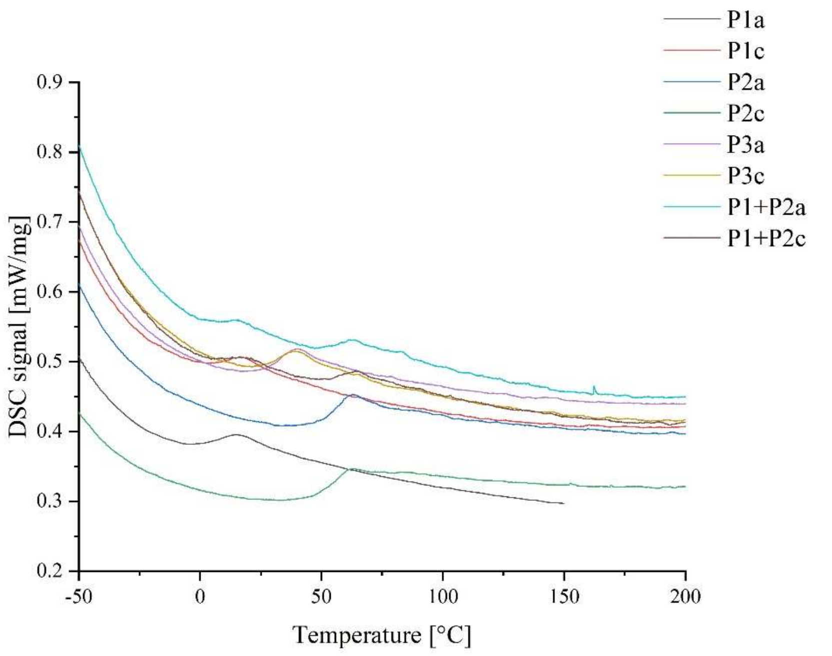

Figure S18. DSC-curves of the third cycle of all polymers with a heating rate of 10 K min^{-1}.

4. Thermogravimetric analysis (TGA)

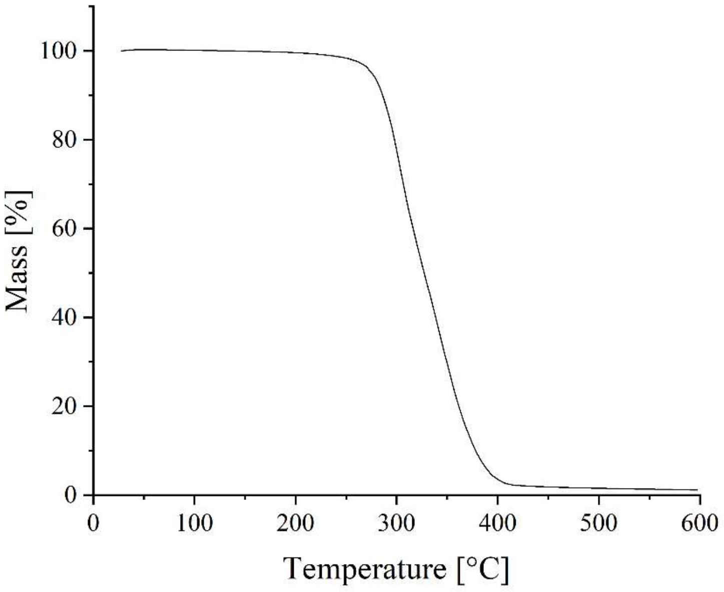

Figure S19. TGA-curves of the polymer **P1a** (heating rate 10 K min^{-1}).

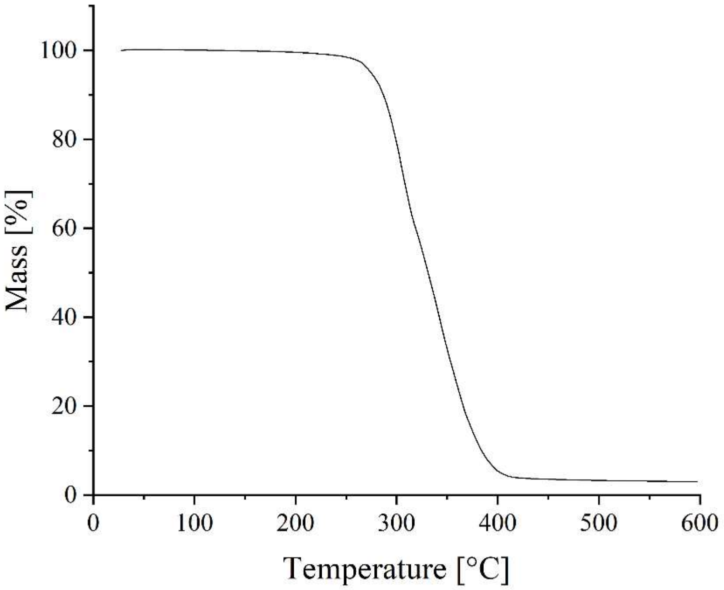

Figure S20. TGA-curves of the polymer **P1c** (heating rate 10 K min^{-1}).

.

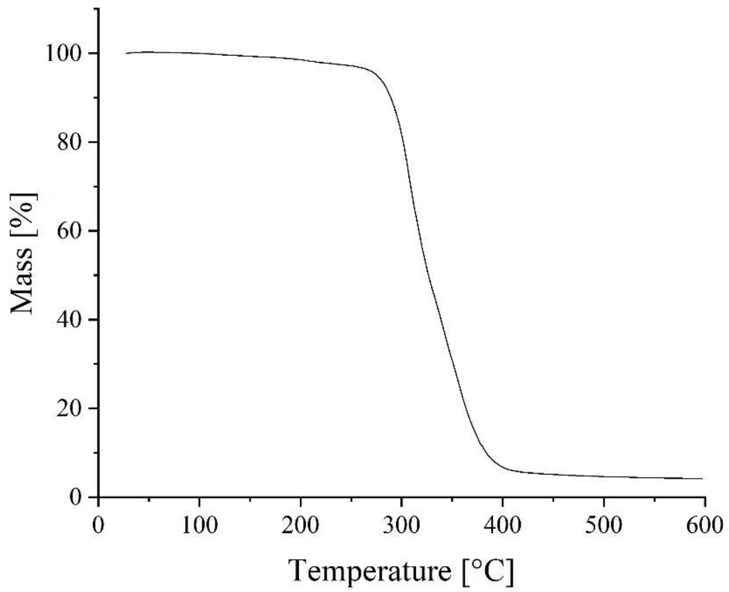

Figure S21. TGA-curves of the polymer **P2a** (heating rate 10 K min^{-1}).

.

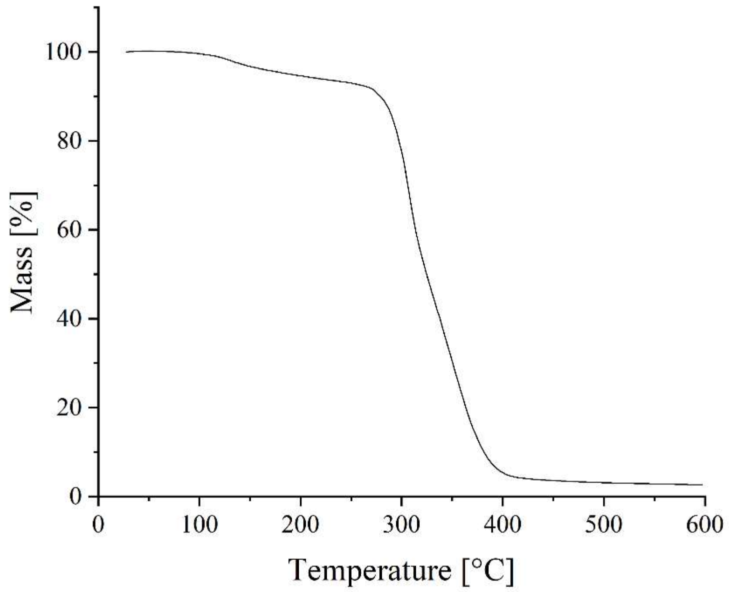

Figure S22. TGA-curves of the polymer **P2c** (heating rate 10 K min^{-1}).

.

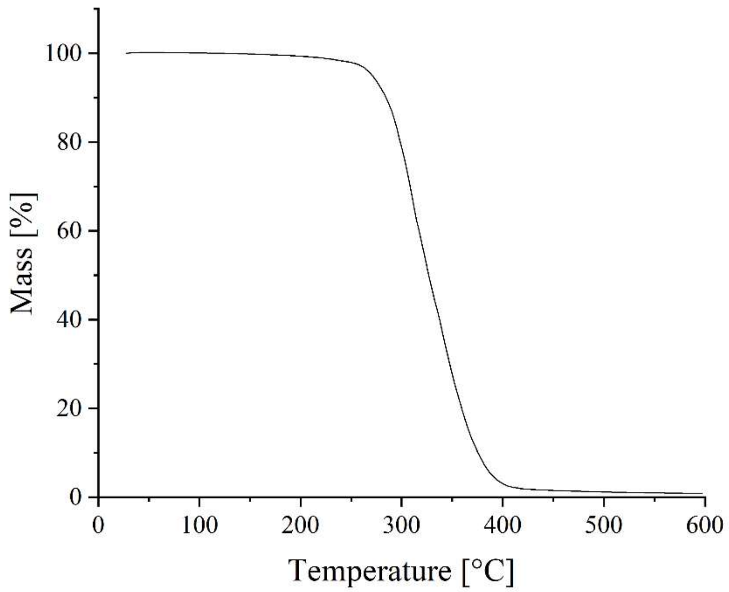

Figure S23. TGA-curves of the polymer **P3a** (heating rate 10 K min^{-1}).

.

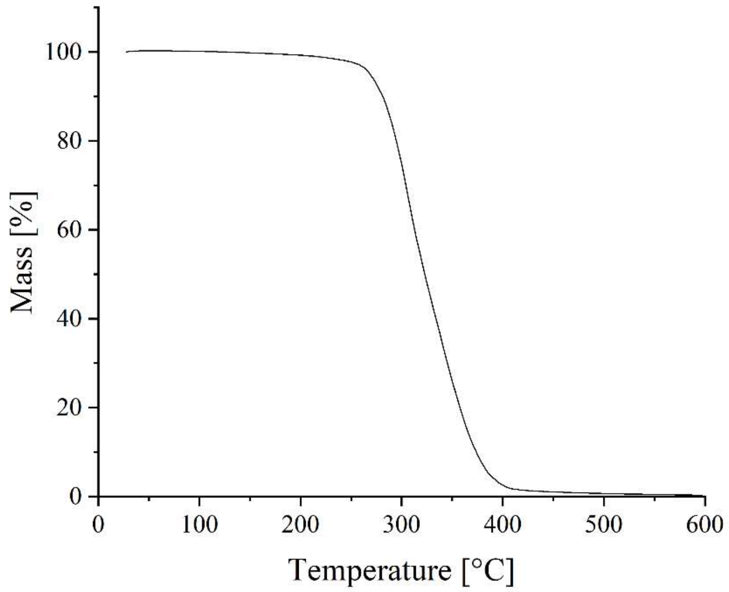

Figure S24. TGA-curves of the polymer **P3c** (heating rate 10 K min^{-1}).

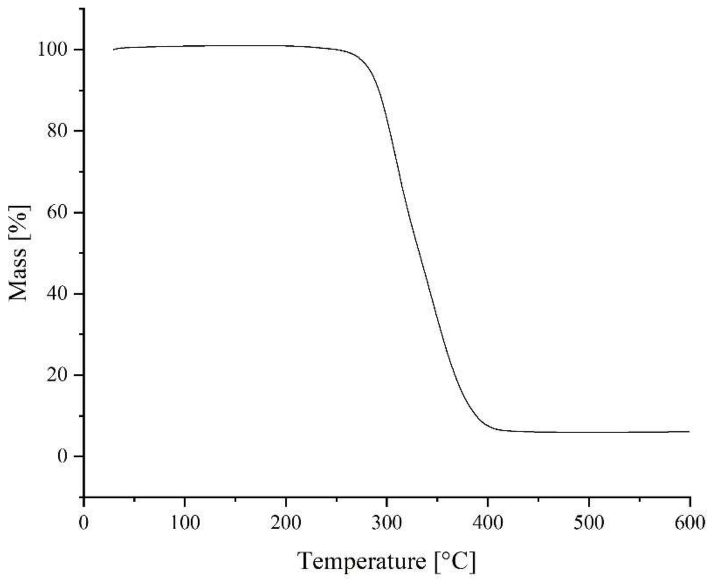

Figure S25. TGA-curves of the polymer **P1+P2a** (heating rate 10 K min^{-1}).

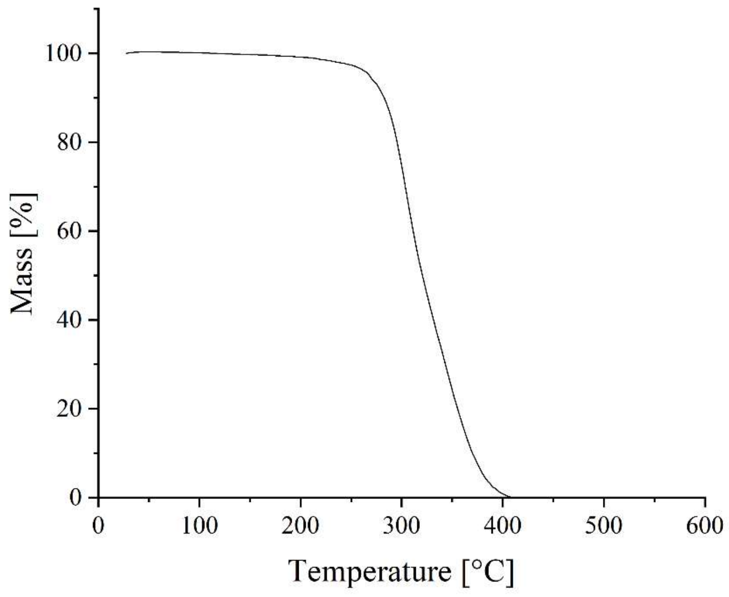

Figure S26. TGA-curves of the polymer **P1+P2c** (heating rate 10 K min^{-1}).

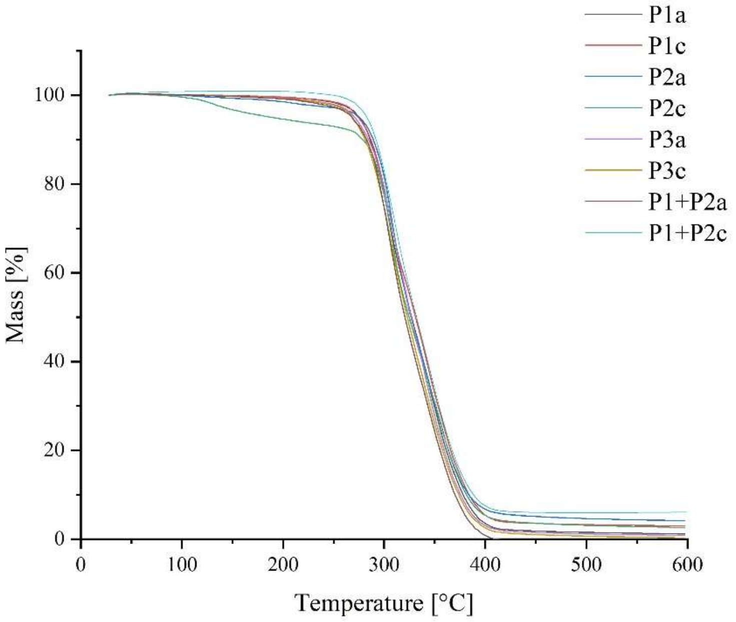

Figure S27. TGA-curves of all polymers (heating rate 10 K min^{-1}).

5. Dynamic mechanical thermal analysis (DMTA)

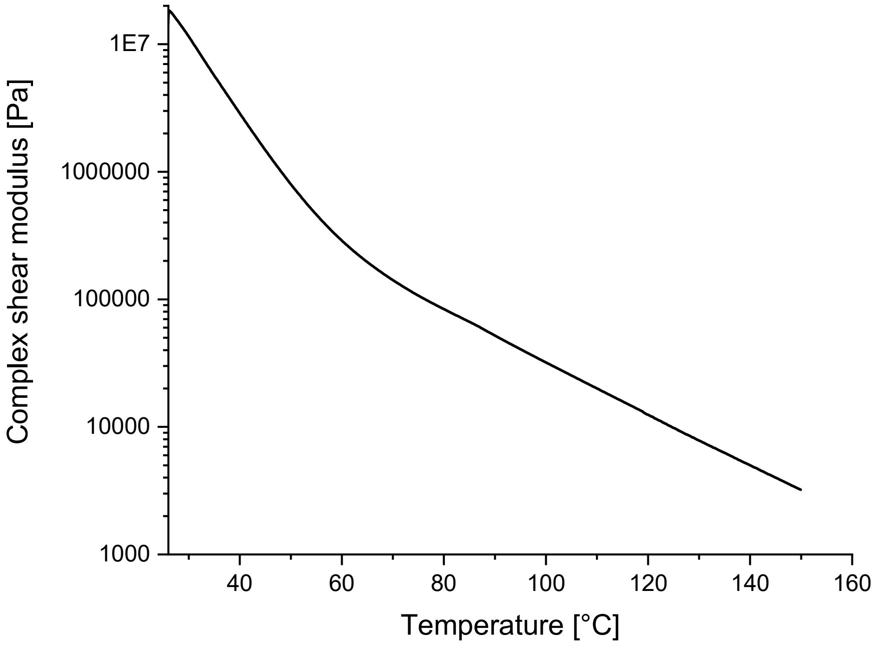

Figure S28. Dynamic mechanical thermal analysis of **P1a**.

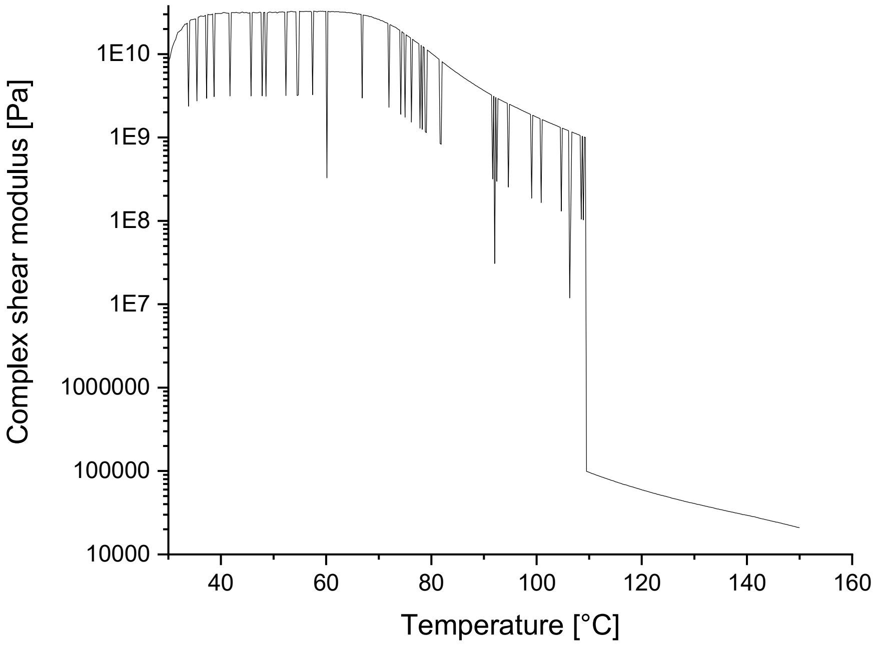

Figure S29. Dynamic mechanical thermal analysis of **P2a** (the results are quite noisy due to bad contact of the polymer to the plates of the rheometer; this behavior could not be improved during further measurements).

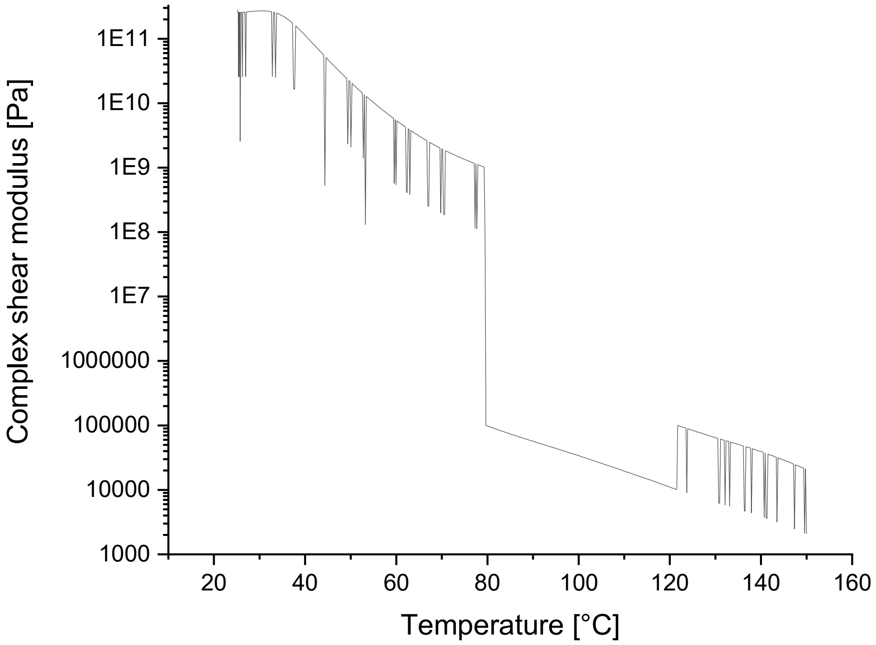

Figure S30. Dynamic mechanical thermal analysis of **P3a** (the results are quite noisy due to bad contact of the polymer to the plates of the rheometer; this behavior could not be improved during further measurements).

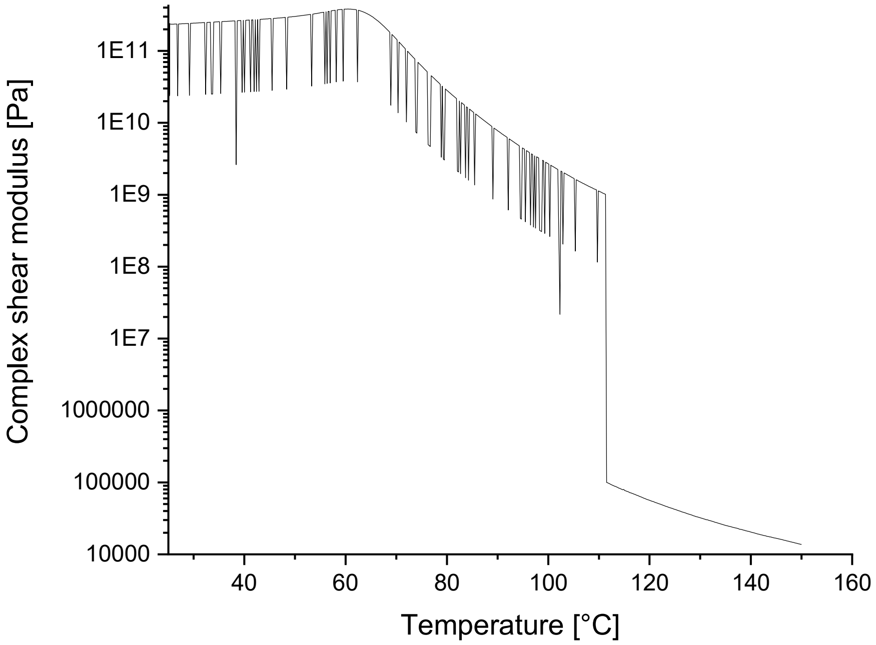

Figure S31. Dynamic mechanical thermal analysis of **P1+P2a** (the results are quite noisy due to bad contact of the polymer to the plates of the rheometer; this behavior could not be improved during further measurements).

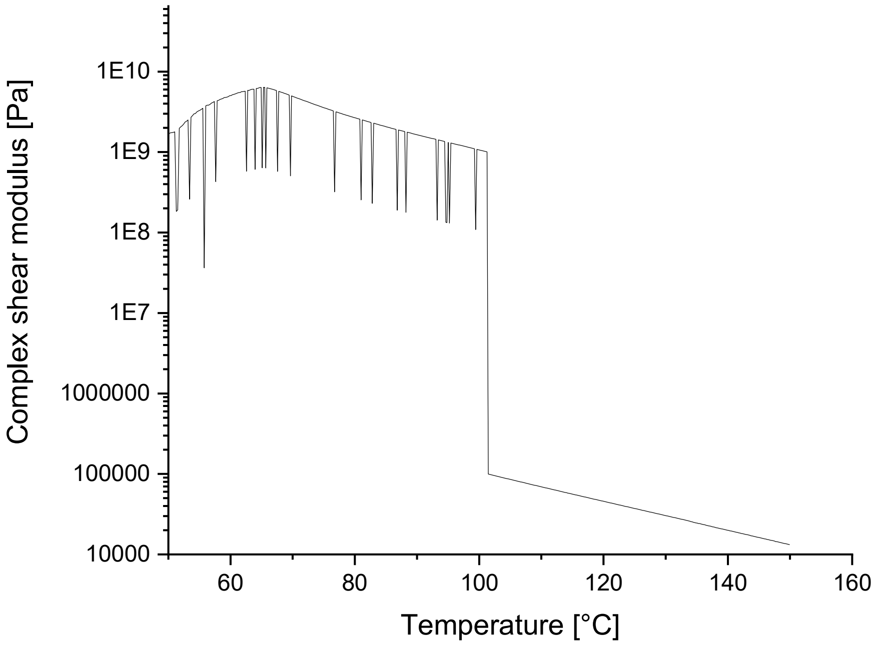

Figure S32. Dynamic mechanical thermal analysis of **P1c** (the results are quite noisy due to bad contact of the polymer to the plates of the rheometer; this behavior could not be improved during further measurements).

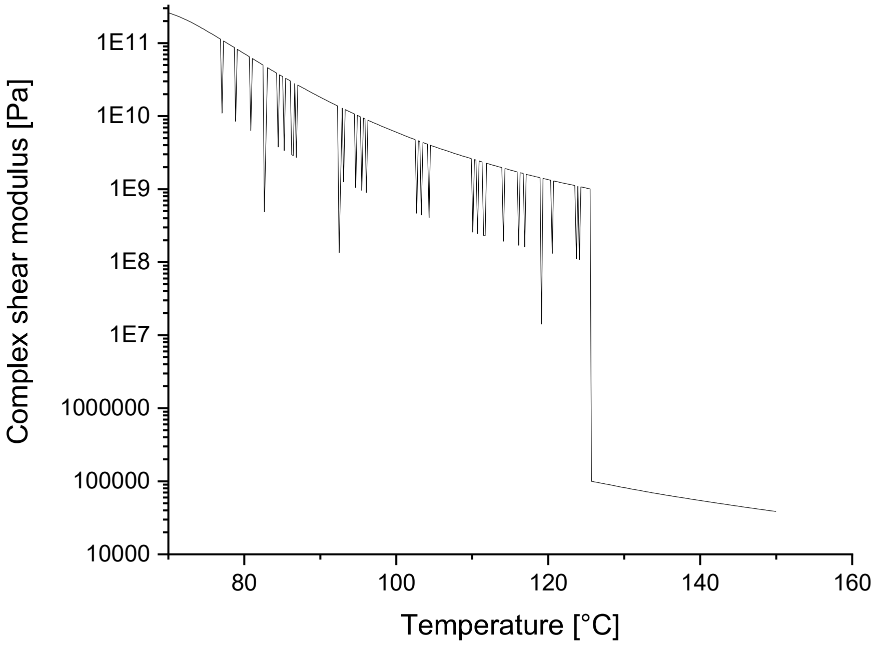

Figure S33. Dynamic mechanical thermal analysis of **P2c** (the results are quite noisy due to bad contact of the polymer to the plates of the rheometer; this behavior could not be improved during further measurements; below 70 °C no contact of the sample to the plates was achieved resulting in no valuable data).

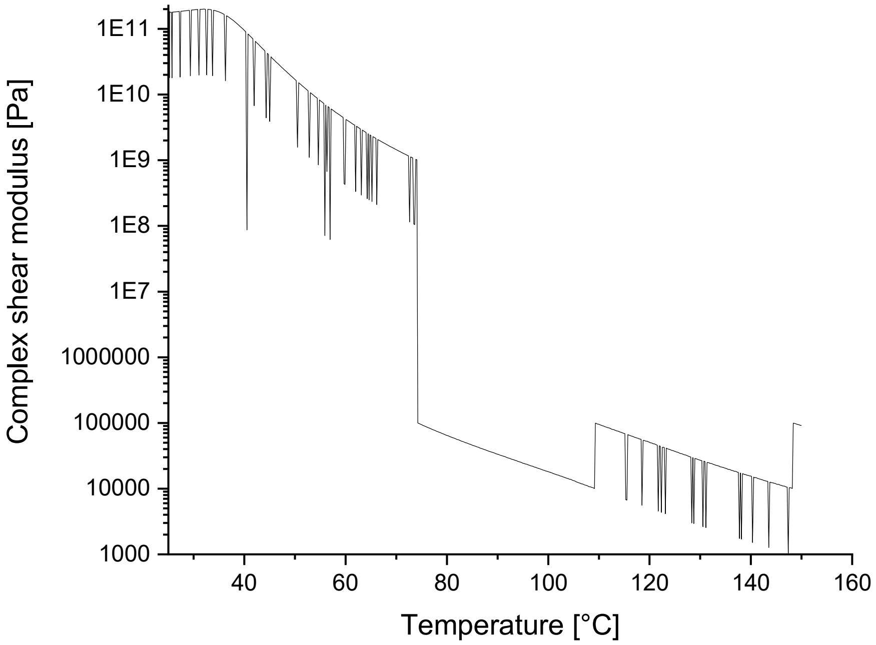

Figure S34. Dynamic mechanical thermal analysis of **P3c** (the results are quite noisy due to bad contact of the polymer to the plates of the rheometer; this behavior could not be improved during further measurements).

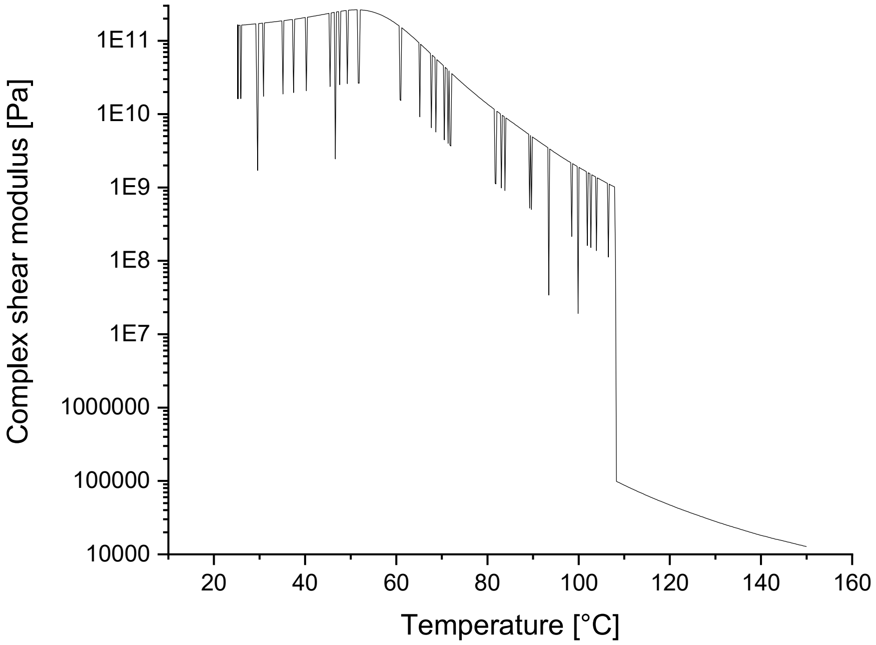

Figure S35. Dynamic mechanical thermal analysis of **P1+P2c** (the results are quite noisy due to bad contact of the polymer to the plates of the rheometer; this behavior could not be improved during further measurements).

6. Photos of the polymer films on paper

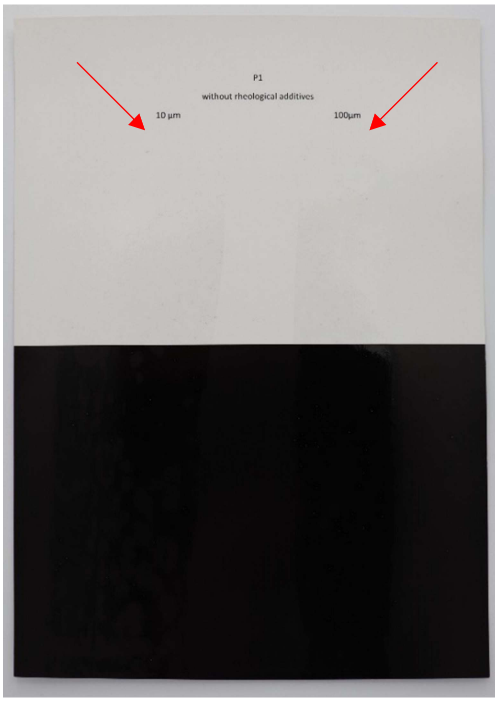

Figure S36. Polymer film of **P1a**. On the left 10 µm wet film thickness and on the right 100 µm.

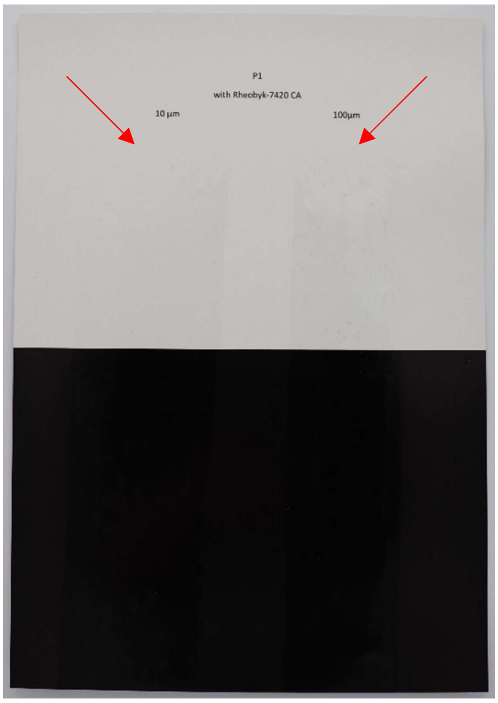

Figure S37. Polymer film of **P1b**. On the left 10 µm wet film thickness and on the right 100 µm.

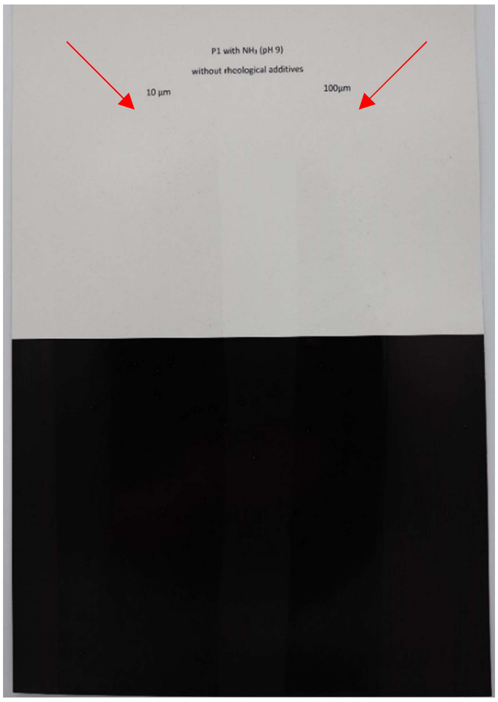

Figure S38. Polymer film of **P1c**. On the left 10 µm wet film thickness and on the right 100 µm.

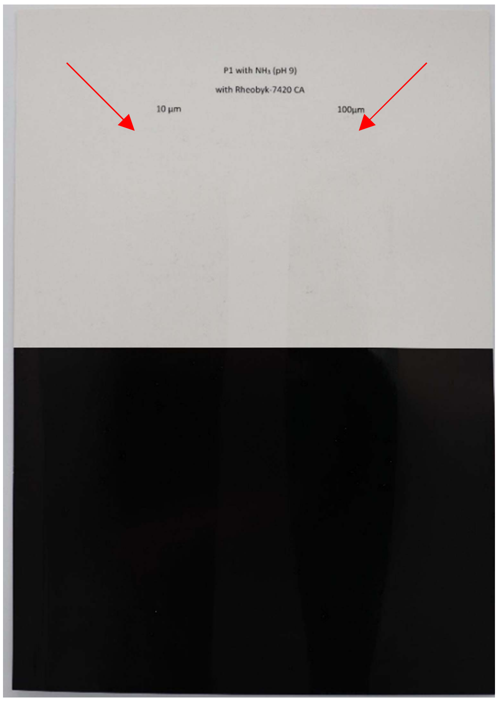

Figure S39. Polymer film of **P1d**. On the left 10 µm wet film thickness and on the right 100 µm.

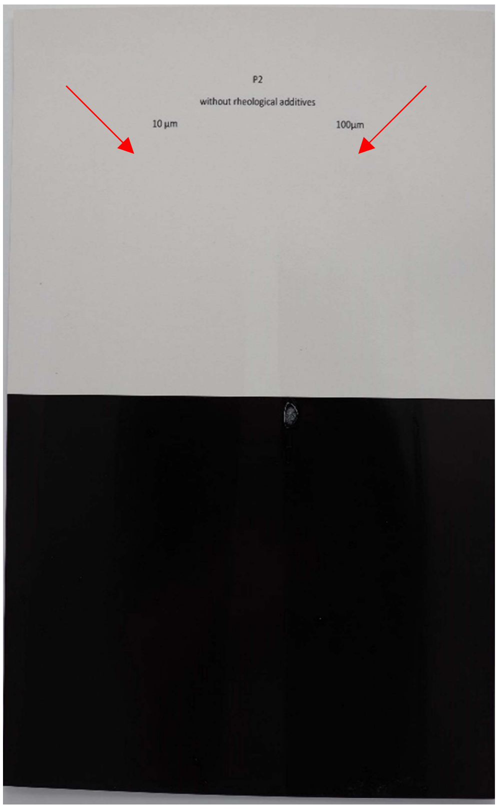

Figure S40. Polymer film of **P2a**. On the left 10 µm wet film thickness and on the right 100 µm.

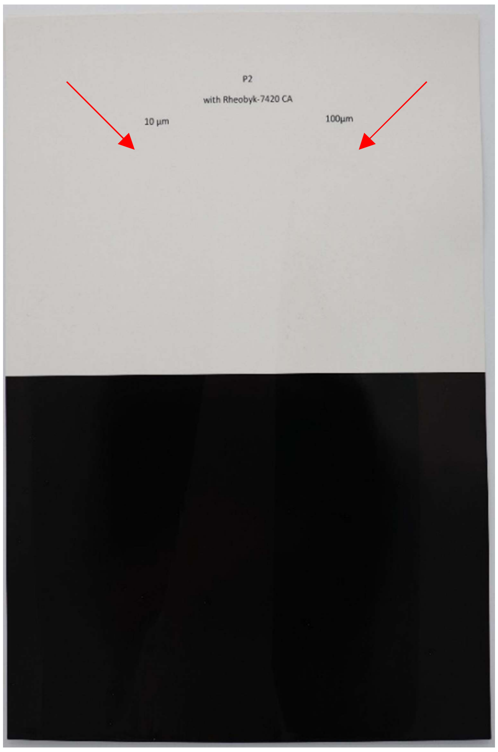

Figure S41. Polymer film of **P2b**. On the left 10 µm wet film thickness and on the right 100 µm.

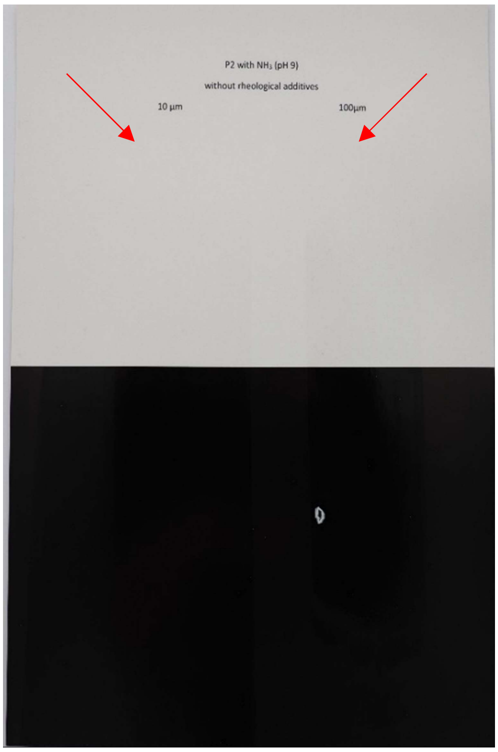

Figure S42. Polymer film of **P2c**. On the left 10 µm wet film thickness and on the right 100 µm.

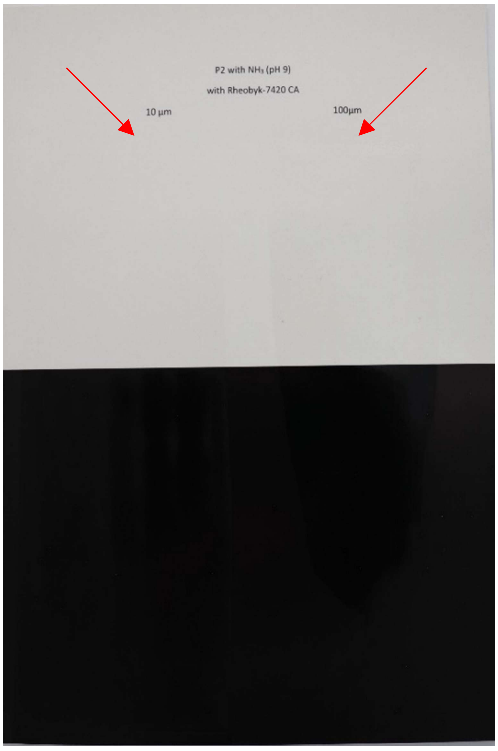

Figure S43. Polymer film of **P2d**. On the left 10 µm wet film thickness and on the right 100 µm.

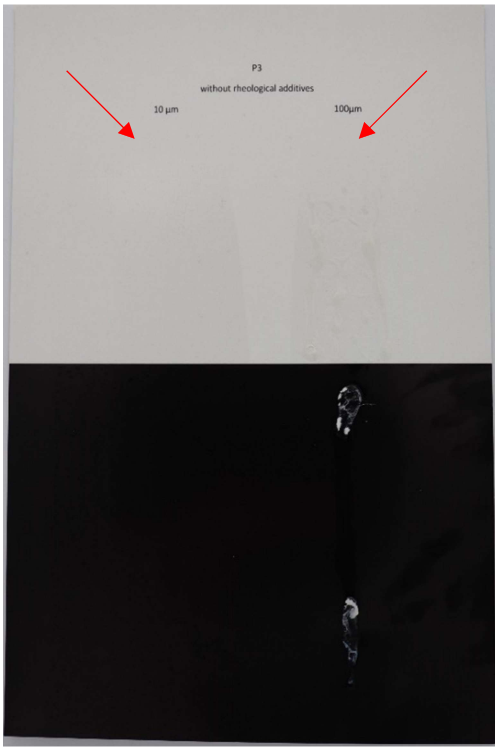

Figure S44. Polymer film of **P3a**. On the left 10 µm wet film thickness and on the right 100 µm. In some places, the top layer of the paper was torn off because the samples were sticky and had been stored in transparent sleeves before documentation (white areas).

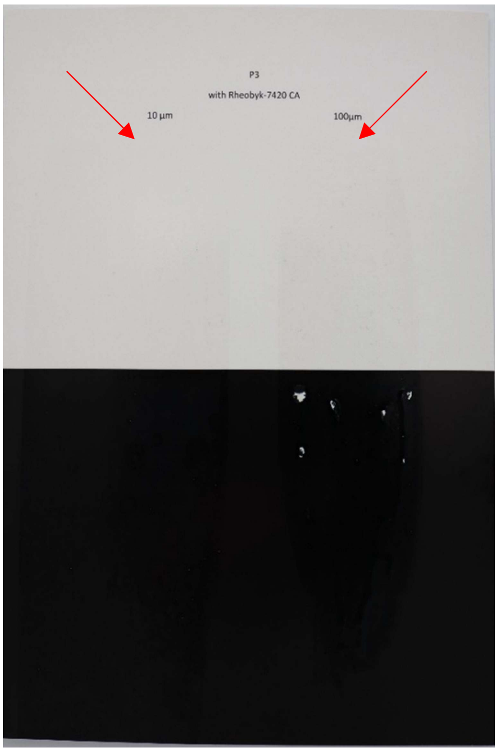

Figure S45. Polymer film of **P3b**. On the left 10 µm wet film thickness and on the right 100 µm. In some places, the top layer of the paper was torn off because the samples were sticky and had been stored in transparent sleeves before documentation (white areas).

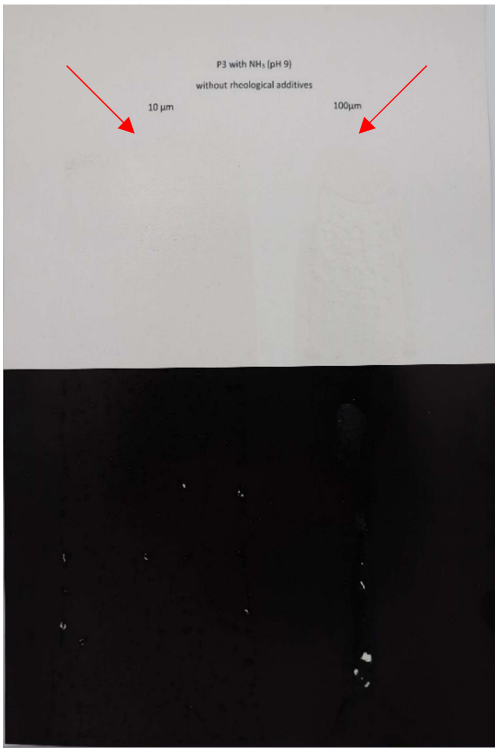

Figure S46. Polymer film of **P3c**. On the left 10 µm wet film thickness and on the right 100 µm. In some places, the top layer of the paper was torn off because the samples were sticky and had been stored in transparent sleeves before documentation (white areas).

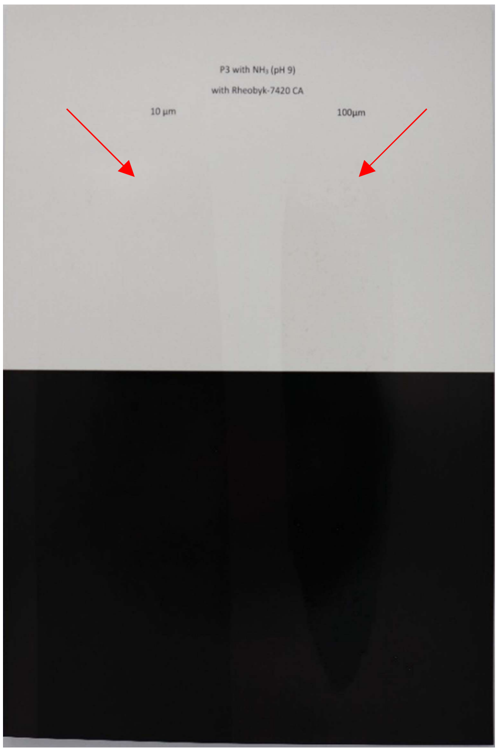

Figure S47. Polymer film of **P3d**. On the left 10 µm wet film thickness and on the right 100 µm.

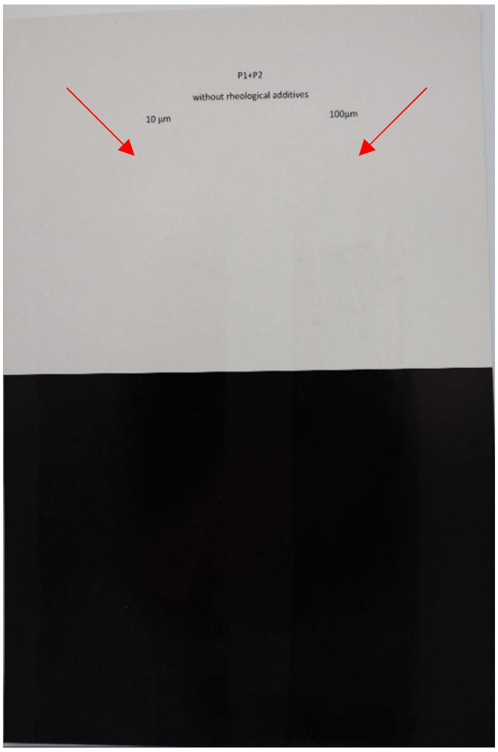

Figure S48. Polymer film of **P1+P2a**. On the left 10 µm wet film thickness and on the right 100 µm.

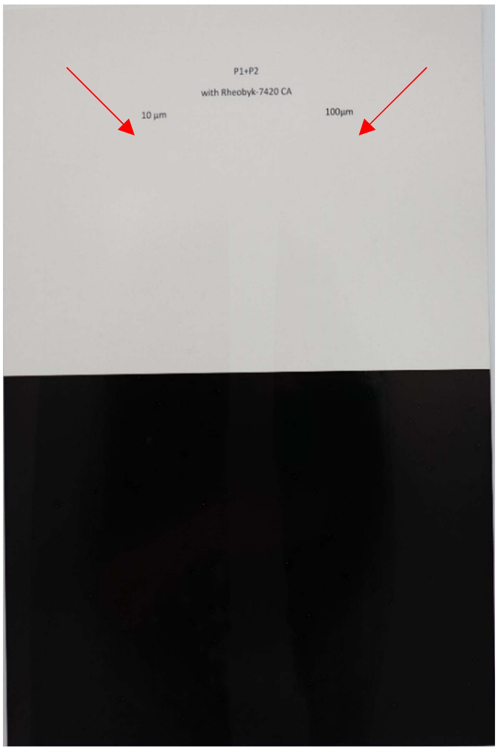

Figure S49. Polymer film of **P1+P2b**. On the left 10 µm wet film thickness and on the right 100 µm.

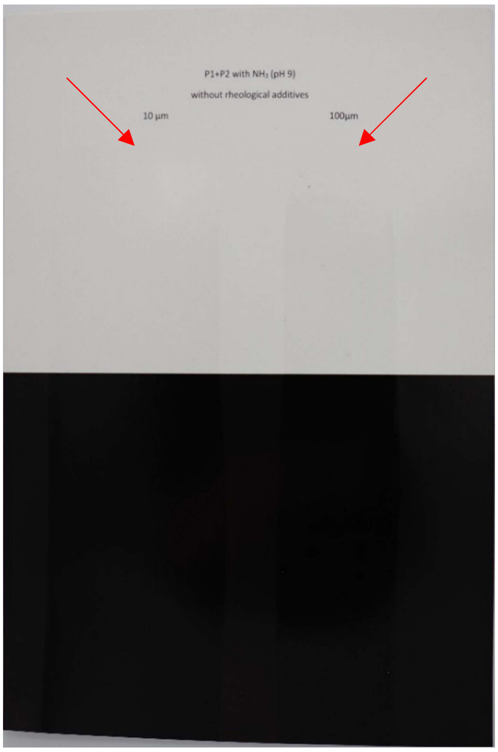

Figure S50. Polymer film of **P1+P2c**. On the left 10 µm wet film thickness and on the right 100 µm.

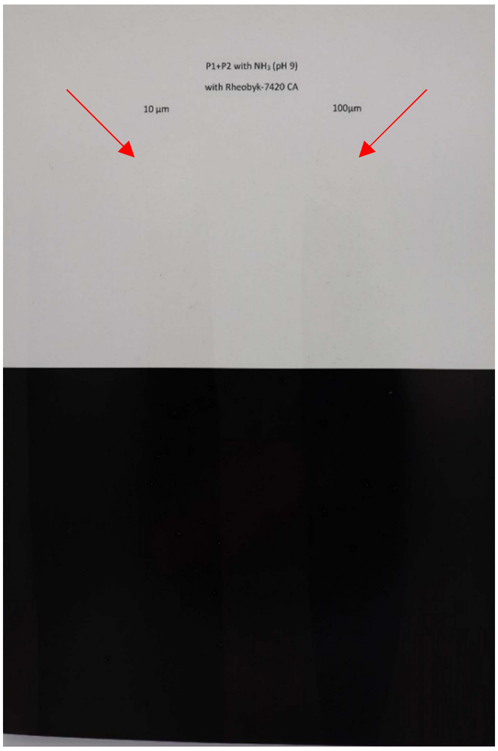

Figure S51. Polymer film of **P1+P2d**. On the left 10 µm wet film thickness and on the right 100 µm.

7. Self-healing of the coatings

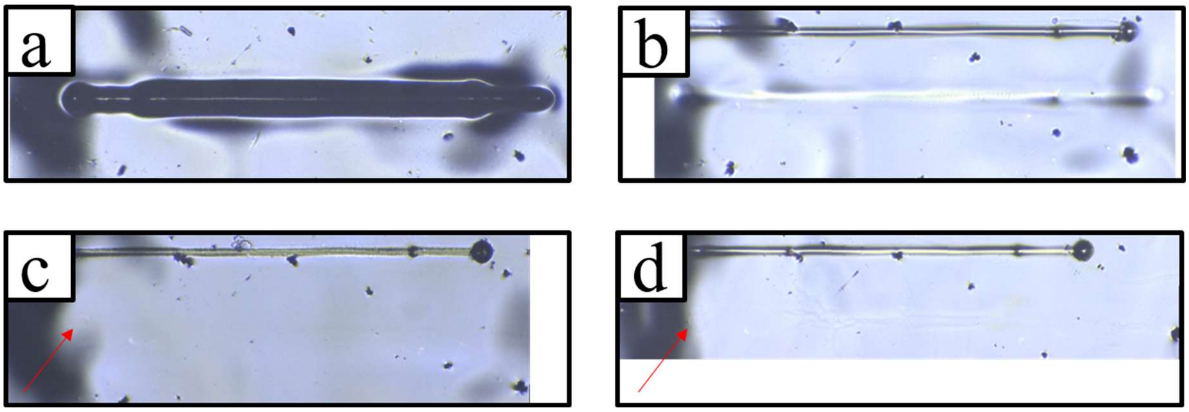

Figure S52. Coating of **P1c** with a layer thickness of 200 μm. Different times of self-healing are shown. In **a**, the crack can be seen in its initial state, in **b** after 15 min, in **c** after 1 h and in **d** after 24 h. The crack is in the middle of the pictures. The crack that can be seen at the top of the picture is caused by taking the picture.

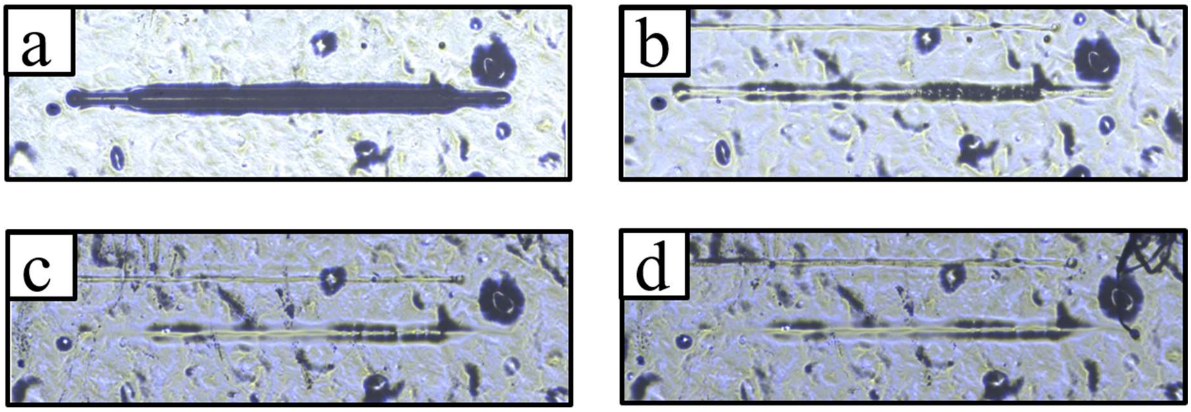

Figure S53. Analysis of the healing behavior of a coating of **P1d** with a layer thickness of 200 μm. Different healing times are shown. In **a**, the crack can be seen in its initial state, in **b** after 15 min, in **c** after 1 h and in **d** after 24 h. The crack is in the middle of the pictures. The crack that can be seen at the top of the picture is caused by taking the picture.

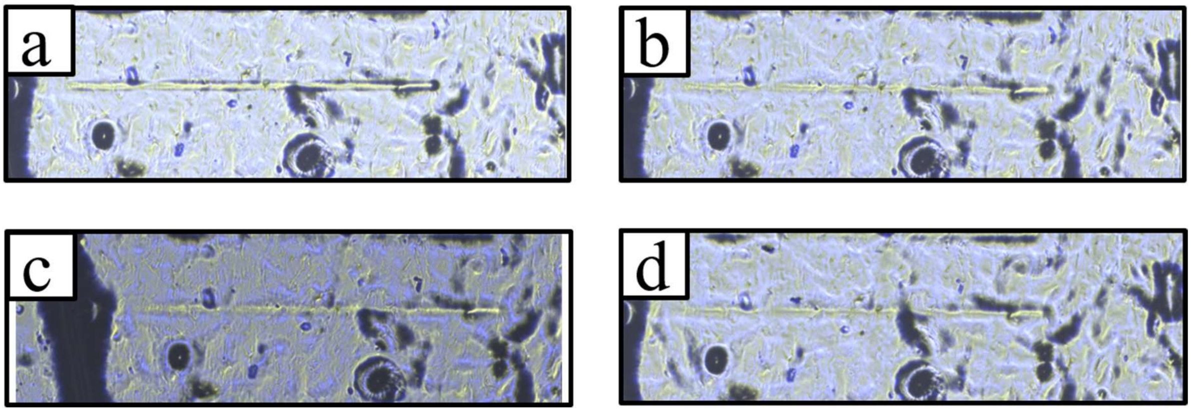

Figure S54. Analysis of the healing behavior of a coating of **P2b** with a layer thickness of 200 µm. Different healing times are shown. In **a**, the crack can be seen in its initial state, in **b** after 15 min, in **c** after 1 h and in **d** after 24 h. The crack is in the middle of the pictures. The crack that can be seen at the top of the picture is caused by taking the picture.

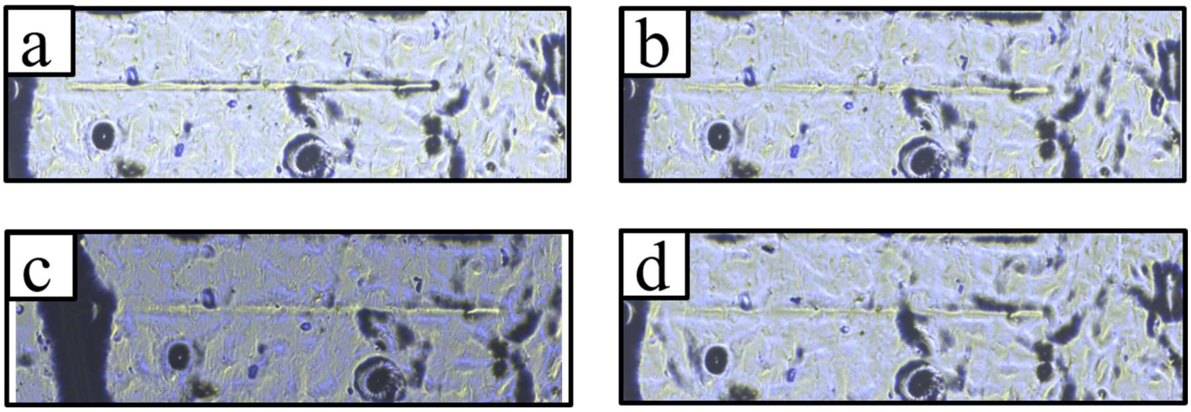

Figure S55. Analysis of the healing behavior of a coating of **P2d** with a layer thickness of 200 µm. Different healing times are shown. In **a**, the crack can be seen in its initial state, in **b** after 15 min, in **c** after 1 h and in **d** after 24 h. The crack is in the middle of the pictures. The crack that can be seen at the top of the picture is caused by taking the picture.

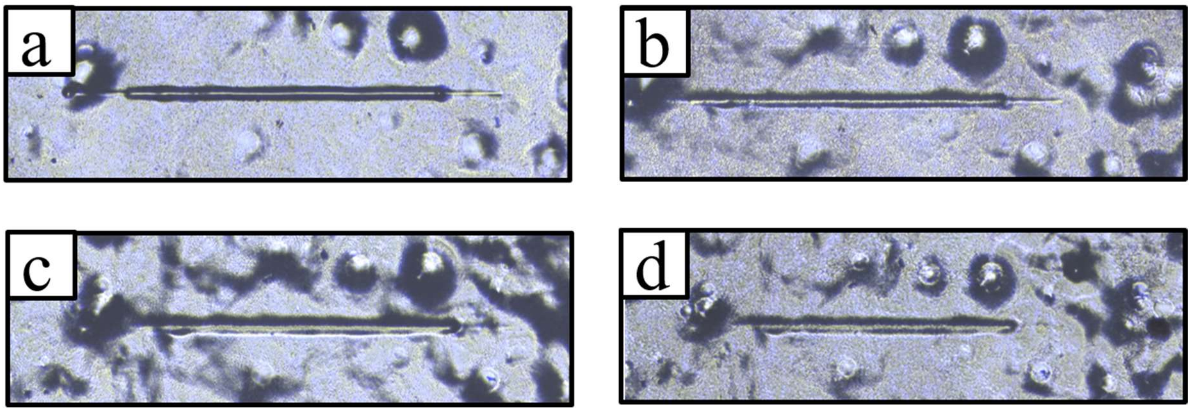

Figure S56. Analysis of the healing behavior of a coating of **P3b** with a layer thickness of 200 µm. Different healing times are shown. In **a**, the crack can be seen in its initial state, in **b** after 15 min, in **c** after 1 h and in **d** after 24 h. The crack is in the middle of the pictures.

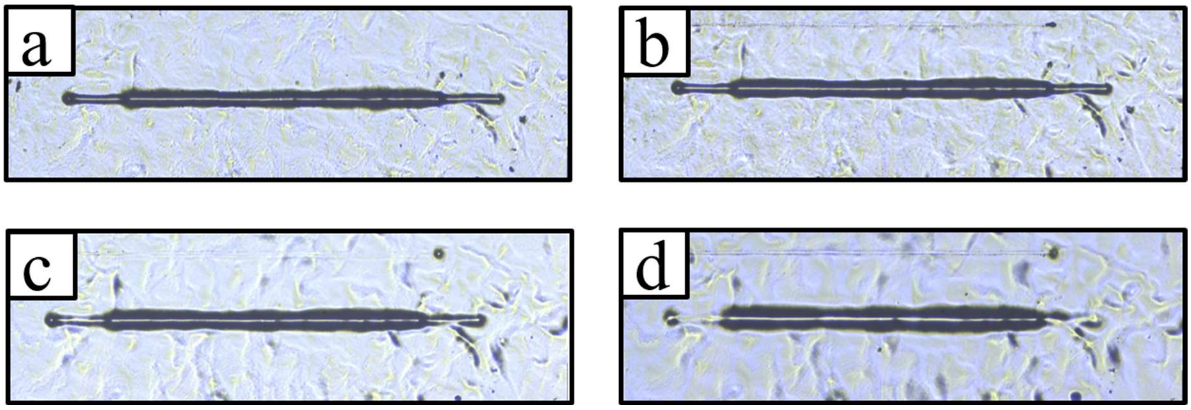

Figure S57. Analysis of the healing behavior of a coating of **P3c** with a layer thickness of 200 µm. Different healing times are shown. In **a**, the crack can be seen in its initial state, in **b** after 15 min, in **c** after 1 h and in **d** after 24 h. The crack is in the middle of the pictures. The crack that can be seen at the top of the picture is caused by taking the picture.

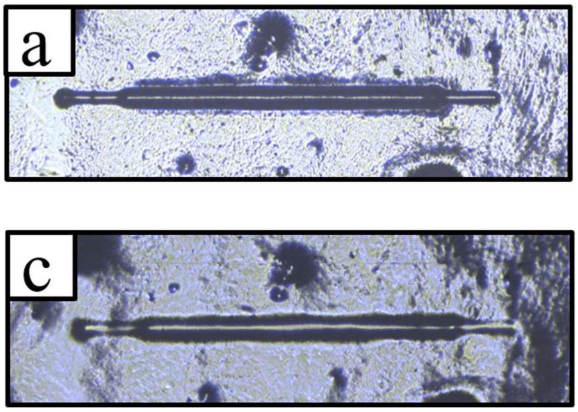

Figure S58. Analysis of the healing behavior of a coating of **P3d** with a layer thickness of 200 µm. Different healing times are shown. In **a**, the crack can be seen in its initial state, in **b** after 15 min, in **c** after 1 h and in **d** after 24 h. The crack is in the middle of the pictures.

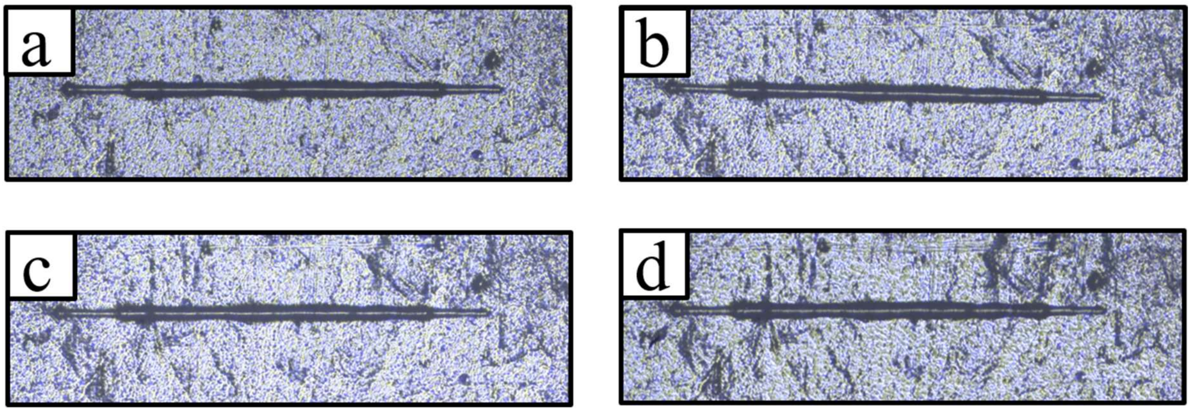

Figure S59. Analysis of the healing behavior of a coating of **P1+P2b** with a layer thickness of 200 µm. Different healing times are shown. In **a**, the crack can be seen in its initial state, in **b** after 15 min, in **c** after 1 h and in **d** after 24 h. The crack is in the middle of the pictures.

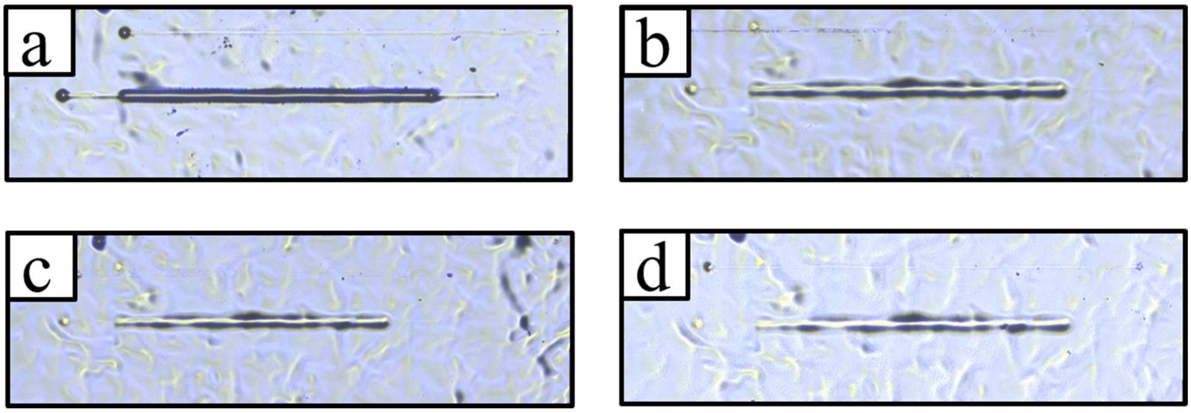

Figure S60. Analysis of the healing behavior of a coating of **P1+P2c** with a layer thickness of 200 µm. Different healing times are shown. In **a**, the crack can be seen in its initial state, in **b** after 15 min, in **c** after 1 h and in **d** after 24 h. The crack is in the middle of the pictures. The crack that can be seen at the top of the picture is caused by taking the picture.

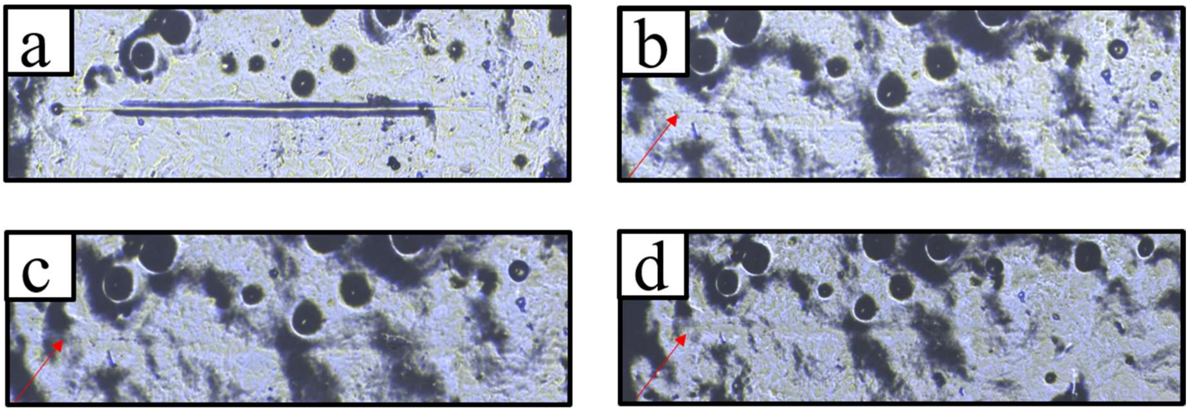

Figure S61. Analysis of the healing behavior of a coating of **P1+P2d** with a layer thickness of 200 µm. Different healing times are shown. In **a**, the crack can be seen in its initial state, in **b** after 15 min, in **c** after 1 h and in **d** after 24 h. The crack is in the middle of the pictures.

8. Self-healing of the bulk

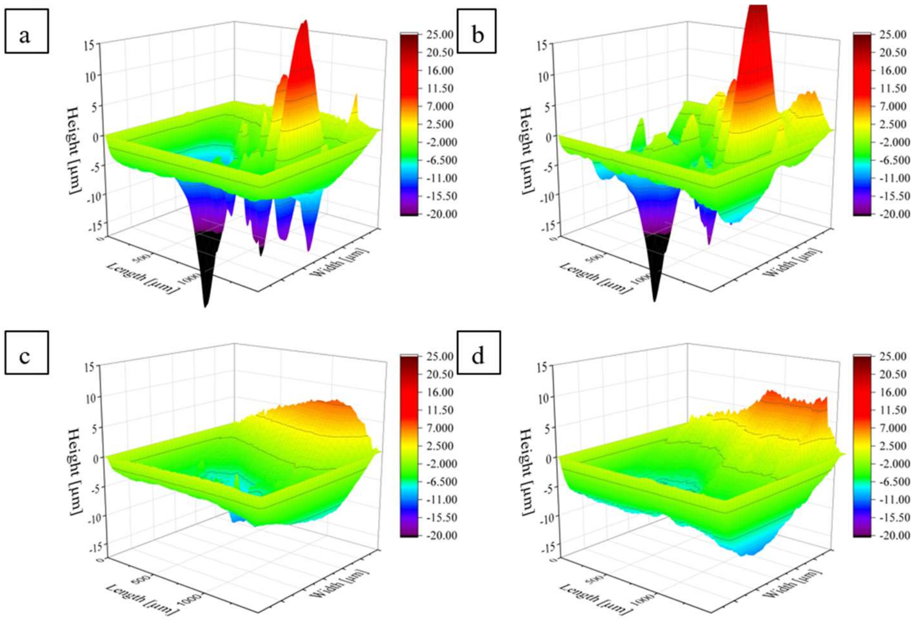

Figure S62. Quantification of the healing behavior of **P1a** featuring 3D-plots of the profile at different times of self-healing. In **a**, the crack can be seen in its initial state, in **b** after 15 min, in **c** after 1 h and in **d** after 24 h.

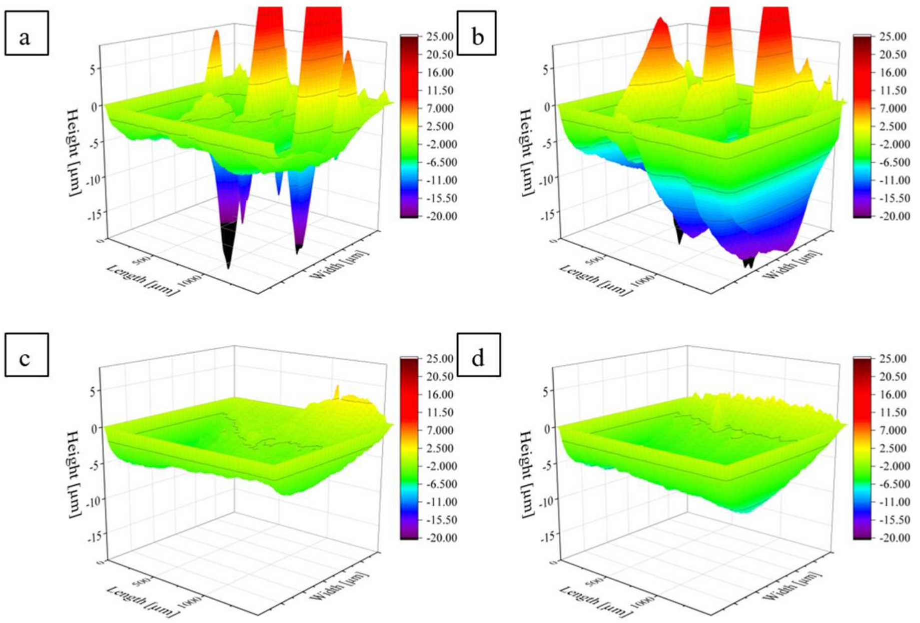

Figure S63. Quantification of the healing behavior of **P1c** featuring 3D-plots of the profile at different times of self-healing. Different times of self-healing are shown. In **a**, the crack can be seen in its initial state, in **b** after 15 min, in **c** after 1 h and in **d** after 24 h.

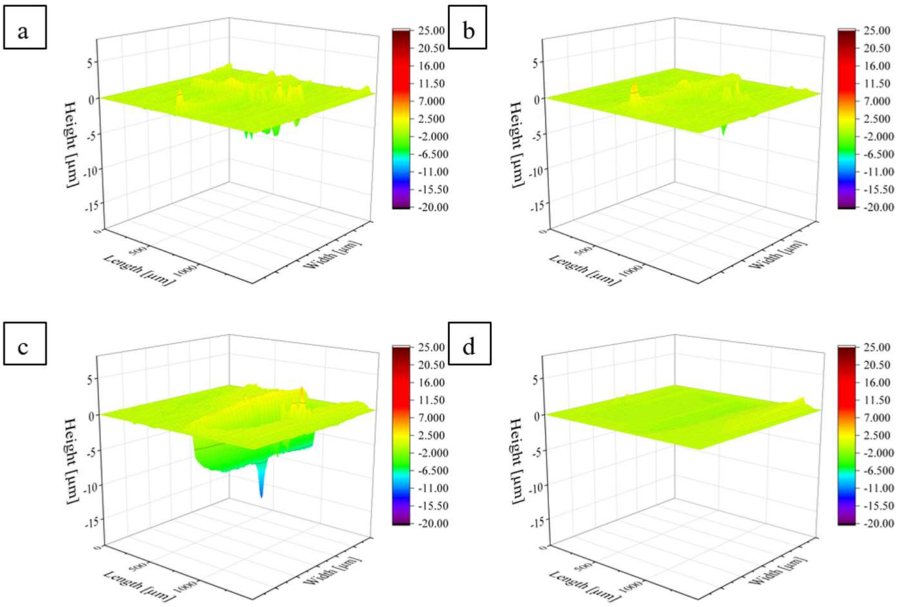

Figure S64. Quantification of the healing behavior of **P2a** featuring 3D-plots of the profile at different times of self-healing. In **a**, the crack can be seen in its initial state, in **b** after 15 min, in **c** after 1 h and in **d** after 24 h.

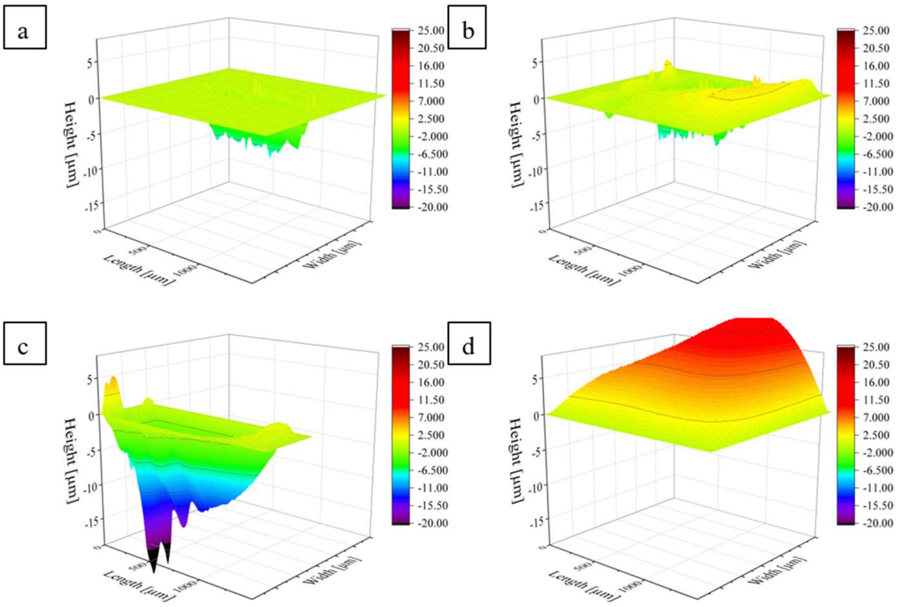

Figure S65. Quantification of the healing behavior of **P2c** featuring 3D-plots of the profile at different times of self-healing. In **a**, the crack can be seen in its initial state, in **b** after 15 min, in **c** after 1 h and in **d** after 24 h.

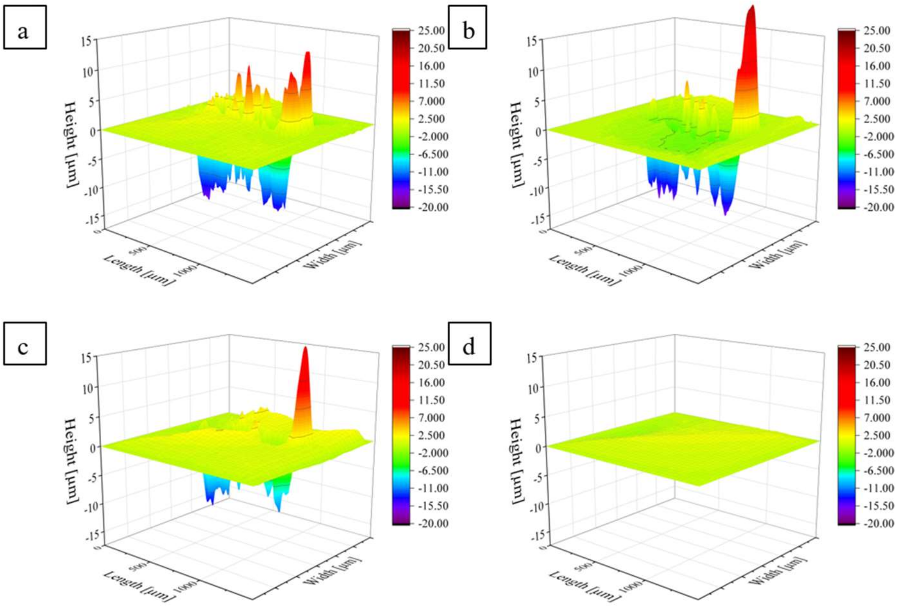

Figure S66. Quantification of the healing behavior of **P3a** featuring 3D-plots of the profile at different times of self-healing. In **a**, the crack can be seen in its initial state, in **b** after 15 min, in **c** after 1 h and in **d** after 24 h.

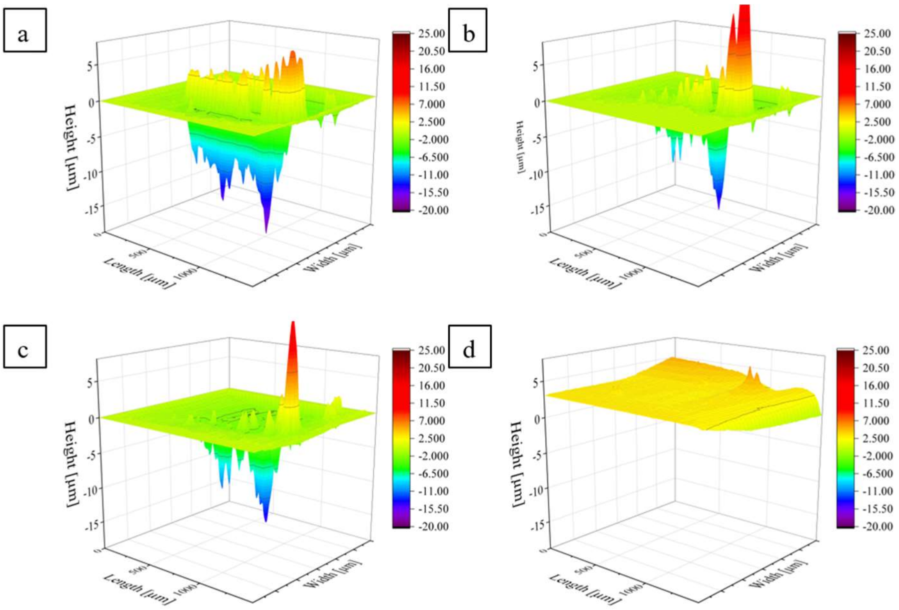

Figure S67. Quantification of the healing behavior of **P3c** featuring 3D-plots of the profile at different times of self-healing. In **a**, the crack can be seen in its initial state, in **b** after 15 min, in **c** after 1 h and in **d** after 24 h.

9. Gloss measurements

Table S1. Gloss measurements with an angle of 20° and a coating thickness of 10 μm (values are normalized on the initial value).

Sample	Before damaging [%]	After damaging [%]	After healing for 15 min [%]	After healing for 1 h [%]
**P1c**	100 ± 3	16 ± 11	40 ± 8	54 ± 6
**P1d**	100 ± 4	55 ± 9	107 ± 11	111 ± 6
**P2b**	100 ± 2	97 ± 5	113 ± 8	120 ± 3
**P2c**	100 ± 10	86 ± 7	111 ± 2	114 ± 7
**P2d**	100 ± 2	79 ± 6	90 ± 7	95 ± 6
**P3b**	100 ± 8	40 ± 7	93 ± 5	105 ± 3
**P3d**	100 ± 3	17 ± 9	49 ± 18	63 ± 5
**P1+P2b**	100 ± 8	53 ± 5	78 ± 4	91 ± 2
**P1+P2c**	100 ± 25	41 ± 19	57 ± 14	57 ± 18
**P1+P2d**	100 ± 2	55 ± 4	73 ± 5	85 ± 6

Table S2. Gloss measurements with an angle of 60° and a coating thickness of 10 µm (values are normalized on the initial value).

Sample	Before damaging [%]	After damaging [%]	After healing for 15 min [%]	After healing for 1 h [%]
**P1c**	100 ± 1	21 ± 13	67 ± 4	78 ± 3
**P1d**	100 ± 1	61 ± 6	99 ± 4	102 ± 3
**P2b**	100 ± 1	82 ± 5	103 ± 6	112 ± 3
**P2c**	100 ± 8	83 ± 8	122 ± 6	124 ± 5
**P2d**	100 ± 1	73 ± 5	95 ± 6	103 ± 6
**P3b**	100 ± 4	40 ± 8	94 ± 4	99 ± 2
**P3d**	100 ± 1	22 ± 11	70 ± 15	81 ± 2
**P1+P2b**	100 ± 3	51 ± 7	84 ± 1	94 ± 1
**P1+P2c**	100 ± 13	42 ± 11	71 ± 11	78 ± 10
**P1+P2d**	100 ± 1	52 ± 4	83 ± 3	91 ± 3

Table S3. Gloss measurements with an angle of 85° and a coating thickness of 10 µm (values are normalized on the initial value).

Sample	Before damaging [%]	After damaging [%]	After healing for 15 min [%]	After healing for 1 h [%]
**P1c**	100 ± 0	39 ± 6	90 ± 1	92 ± 1
**P1d**	100 ± 2	72 ± 2	96 ± 1	97 ± 1
**P2b**	100 ± 8	78 ± 6	98 ± 7	104 ± 4
**P2c**	100 ± 34	95 ± 27	151 ± 9	136 ± 10
**P2d**	100 ± 1	74 ± 3	91 ± 4	95 ± 6
**P3b**	100 ± 1	52 ± 5	96 ± 2	96 ± 2
**P3d**	100 ± 1	38 ± 10	87 ± 12	95 ± 2
**P1+P2b**	100 ± 1	50 ± 6	95 ± 1	98 ± 1
**P1+P2c**	100 ± 14	39 ± 7	91 ± 5	96 ± 5
**P1+P2d**	100 ± 0	51 ± 4	91 ± 1	94 ± 1

Table S4. Gloss measurements with an angle of 20° and a coating thickness of 100 μm (values are normalized on the initial value).

Sample	Before damaging [%]	After damaging [%]	After healing for 15 min [%]	After healing for 1 h [%]
**P1c**	100 ± 6	20 ± 13	91 ± 4	104 ± 4
**P1d**	100 ± 1	31 ± 13	93 ± 5	93 ± 5
**P2d**	100 ± 3	50 ± 2	68 ± 3	72 ± 2
**P3b**	100 ± 4	24 ± 8	85 ± 7	85 ± 4
**P3d**	100 ± 5	20 ± 8	76 ± 12	82 ± 13
**P1+P2b**	100 ± 4	56 ± 13	93 ± 13	114 ± 14
**P1+P2c**	100 ± 5	29 ± 13	43 ± 11	51 ± 8
**P1+P2d**	100 ± 3	47 ± 5	88 ± 2	105 ± 2

Table S5. Gloss measurements with an angle of 60° and a coating thickness of 100 µm (values are normalized on the initial value).

Sample	Before damaging [%]	After damaging [%]	After healing for 15 min [%]	After healing for 1 h [%]
**P1c**	100 ± 3	24 ± 14	96 ± 1	101 ± 1
**P1d**	100 ± 2	39 ± 10	97 ± 3	97 ± 3
**P2d**	100 ± 1	59 ± 3	80 ± 1	84 ± 1
**P3b**	100 ± 2	32 ± 4	88 ± 3	89 ± 2
**P3d**	100 ± 2	32 ± 7	80 ± 5	82 ± 7
**P1+P2b**	100 ± 2	49 ± 14	85 ± 9	97 ± 10
**P1+P2c**	100 ± 2	33 ± 11	68 ± 6	75 ± 3
**P1+P2d**	100 ± 1	48 ± 3	93 ± 1	106 ± 1

Table S6. Gloss measurements with an angle of 85° and a coating thickness of 100 µm (values are normalized on the initial value).

Sample	Before damaging [%]	After damaging [%]	After healing for 15 min [%]	After healing for 1 h [%]
**P1c**	100 ± 2	46 ± 6	99 ± 1	101 ± 2
**P1d**	100 ± 2	67 ± 4	97 ± 2	97 ± 2
**P2d**	100 ± 2	77 ± 4	89 ± 0	91 ± 1
**P3b**	100 ± 1	42 ± 7	75 ± 2	79 ± 3
**P3d**	100 ± 2	44 ± 8	74 ± 5	79 ± 3
**P1+P2b**	100 ± 2	43 ± 17	85 ± 9	84 ± 17
**P1+P2c**	100 ± 1	37 ± 9	91 ± 6	92 ± 2
**P1+P2d**	100 ± 1	48 ± 3	94 ± 1	96 ± 2

10. Barrier properties of the coatings against fat

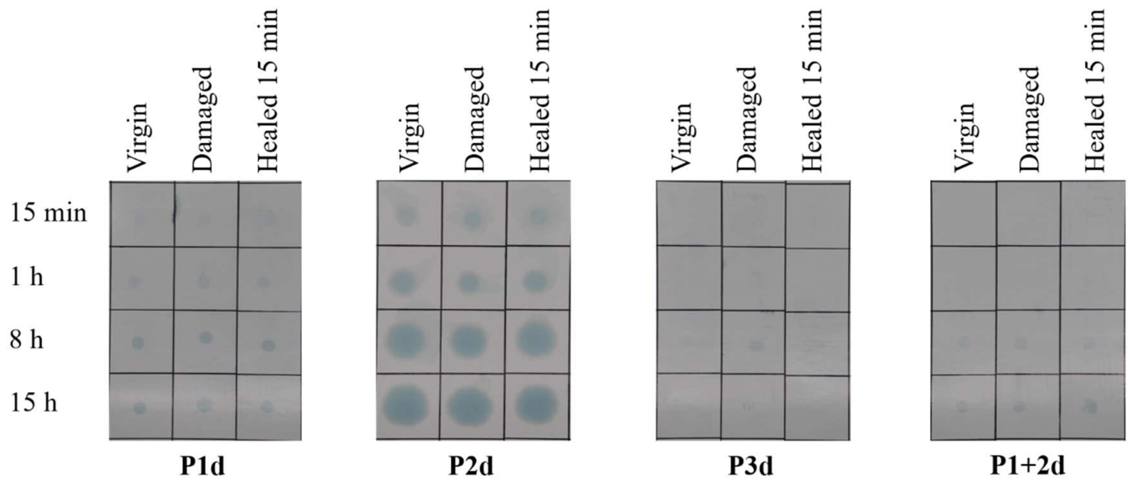

Figure S67. Barrier properties of the coatings against fat. The films are divided into grids from left to right is the undamaged film, the damaged film and the film after 15 min curing at 100 °C. From top to bottom, the time dependency of the penetration in the paper is shown after 15 min, 1 h, 8 h and 15 h.

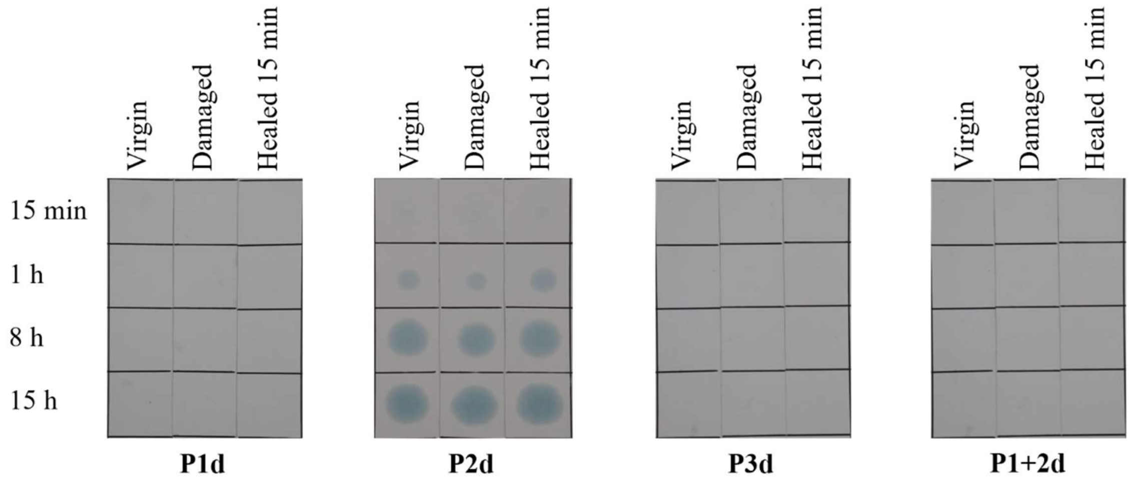

Figure S68. Barrier properties of the coatings against fat, showing the back of the paper. The films are divided into grids from left to right is the undamaged film, the damaged film and the film after 15 min curing at 100 °C. From top to bottom, the time dependency of the penetration in the paper is shown after 15 min, 1 h, 8 h and 15 h.

Table S7. Colored percentage of the surface of the paper with a coating of **P1d** with a film thickness of 10 µm during the testing of the barrier properties of the coatings against fat.

Application time	Colored percentage of the surface [%]		
	Virgin	Damaged	Healed 15 min at 100 °C
15 min	3	4	3
1 h	3	3	3
8 h	3	3	3
15 h	3	4	3

Table S8. Colored percentage of the surface of the paper with a coating of **P2d** with a film thickness of 10 µm during the testing of the barrier properties of the coatings against fat.

Application time	Colored percentage of the surface [%]		
	Virgin	Damaged	Healed 15 min at 100 °C
15 min	8	6	5
1 h	20	15	16
8 h	35	35	38
15 h	45	42	41

Table S9. Colored percentage of the reverse side of the paper with a coating of **P2d** with a film thickness of 10 µm during the testing of the barrier properties of the coatings against fat.

**Application time**	**Colored percentage of the surface [%]**		
	**Virgin**	**Damaged**	**Healed 15 min at 100 °C**
15 min	0	0	0
1 h	9	8	13
8 h	28	26	31
15 h	35	37	39

Table S10. Colored percentage of the surface of the paper with a coating of **P3d** with a film thickness of 10 µm during the testing of the barrier properties of the coatings against fat.

**Application time**	**Colored percentage of the surface [%]**		
	**Virgin**	**Damaged**	**Healed 15 min at 100 °C**
15 min	0	0	0
1 h	0	0	0
8 h	0	3	0
15 h	0	1	0

Table S11. Colored percentage of the surface of the paper with a coating of **P1+P2d** with a film thickness of 10 μm during the testing of the barrier properties of the coatings against fat.

**Application time**	**Colored percentage of the surface [%]**		
	**Virgin**	**Damaged**	**Healed 15 min at 100 °C**
15 min	0	0	0
1 h	0	0	0
8 h	3	3	1
15 h	3	3	4
